# Molecular basis for intestinal mucin recognition by galectin-3 and C-type lectins

**DOI:** 10.1096/fj.201700619R

**Published:** 2018-01-29

**Authors:** Charlotte Leclaire, Karine Lecointe, Patrick A. Gunning, Sandra Tribolo, Devon W. Kavanaugh, Alexandra Wittmann, Dimitrios Latousakis, Donald A. MacKenzie, Norihito Kawasaki, Nathalie Juge

**Affiliations:** Quadram Institute Bioscience, Norwich Research Park, Norwich, United Kingdom

**Keywords:** mucus, *O*-glycosylation, *N*-glycosylation, immune system

## Abstract

Intestinal mucins trigger immune responses upon recognition by dendritic cells *via* protein–carbohydrate interactions. We used a combination of structural, biochemical, biophysical, and cell-based approaches to decipher the specificity of the interaction between mucin glycans and mammalian lectins expressed in the gut, including galectin (Gal)-3 and C-type lectin receptors. Gal-3 differentially recognized intestinal mucins with different *O*-glycosylation profiles, as determined by mass spectrometry (MS). Modification of mucin glycosylation, *via* chemical treatment leading to a loss of terminal glycans, promoted the interaction of Gal-3 to poly-*N*-acetyllactosamine. Specific interactions were observed between mucins and mouse dendritic cell-associated lectin (mDectin)-2 or specific intercellular adhesion molecule–grabbing nonintegrin-related-1 (SIGN-R1), but not mDectin-1, using a cell-reporter assay, as also confirmed by atomic force spectroscopy. We characterized the *N*-glycosylation profile of mouse colonic mucin (Muc)-2 by MS and showed that the interaction with mDectin-2 was mediated by high-mannose *N*-glycans. Furthermore, we observed Gal-3 binding to the 3 C-type lectins by force spectroscopy. We showed that mDectin-1, mDectin-2, and SIGN-R1 are decorated by *N*-glycan structures that can be recognized by the carbohydrate recognition domain of Gal-3. These findings provide a structural basis for the role of mucins in mediating immune responses and new insights into the structure and function of major mammalian lectins.—Leclaire, C., Lecointe, K., Gunning, P. A., Tribolo, S., Kavanaugh, D. W., Wittmann, A., Latousakis, D., MacKenzie, D. A., Kawasaki, N., Juge, N. Molecular basis for intestinal mucin recognition by galectin-3 and C-type lectins.

The mucosal immune system comprises the largest part of the entire immune system. In the gastrointestinal (GI) tract, goblet cells (GCs) have an established role in innate immunity by secreting mucins, a major component of the mucus layer (1, 2), which provides a first line of defense against physical and chemical injury and protects against invasion by pathogens (3, 4). All mucins are characterized by mucin domains rich in Ser, Thr, and Pro amino acid residues, which are the site of heavy *O*-glycosylation. MUC2, -5AC, -6, and -5B are the main gel-forming mucins secreted by GCs in the intestine, stomach surface, stomach glands, and salivary glands, respectively (4). In addition to the central mucin domains, gel-forming mucins have N- and C-terminal domains that are involved in mucin dimerization, oligomerization, polymerization, and the formation of the protective mucus gels (5). The intestinal mucus is built around the large highly glycosylated gel-forming MUC2 (Muc2 in mouse) (6, 7). In the small intestine (SI), a loose and penetrable mucus layer allows smooth transport of nutrients while reducing mucosal exposure to damaging agents. In the large intestine, mucus shows a bilayer organization with the outer mucus layer providing a natural habitat for the commensal bacteria, whereas the stratified inner mucus layer restricts bacterial access to the epithelium (8, 9). The delivery of luminal substances across the intestinal mucus and epithelium to the immune system is a critical event in immune surveillance, resulting in tolerance to dietary antigens and immunity to pathogens. Recently, GC-associated passages were identified as a pathway delivering luminal antigens to underlying lamina propria dendritic cells (DCs) in the steady state (10). The mechanism of this uptake is not fully understood (11), but it is believed to be coupled to mucin secretion (12) and involves sentinel GCs localized to the colonic crypt entrance (13).

The glycan structures present in mucins are diverse and complex and consist of 4 core mucin-type *O*-glycans containing *N*-acetylgalactosamine (GalNAc), galactose and *N*-acetylglucosamine (GlcNAc). Mucin *O*-glycosylation starts in the Golgi apparatus with the attachment of GalNAc residues to the hydroxyl group of Ser or Thr of the protein backbone to form the Tn antigen (GalNAcα1-Ser/Thr). This glycan is then elongated into core-1 [Galβ1-3GalNAcα1-Ser/Thr, also known as Thomsen-Friedenreich- (TF-), or T-antigen], core-2 (Galβ1-3(GlcNAcβ1-6)GalNAcα1-Ser/Thr), core-3 (GlcNAcβ1-3GalNAcα1-Ser/Thr), or core-4 [GlcNAcβ1-3(GlcNAcβ1-6)GalNAcα1-Ser/Thr] structures (14). Core-1– and -3–derived mucin-type *O*-linked oligosaccharides (*O*-glycans) are major components of the colonic mucus layer (15–17). These core structures may be further elongated by the addition of other carbohydrates [*e.g.*, *N*-acetyllactosamine (LacNAc)] or terminally modified by fucose, sialic acid, or sulfate groups. *O*-glycans can exhibit a remarkable heterogeneity and diversity, as mucins can also carry ABO or Lewis-type histo-blood group epitopes (18). The ratio of sialic acid to fucose increases along the GI tract, from the ileum to the distal colon in humans (17), whereas an inverse gradient is found in mice (19). Charged glycans such as the Sd/CAD epitope [GalNAcβ1–4(NeuAcα2–3)Galβ1−] are more predominant in the human distal colon, whereas fucosylated glycans (ABO histo-blood group antigens) are more abundant in the proximal parts of the intestine (16). Abrogation of mucin *O*-glycans has highlighted the role of specific glycan chains of mucins in maintaining mucosal barrier function (20). For example, mice lacking core-3 β1,3-*N*-acetylglucosaminyltransferase (C3GnT), an enzyme involved in the biosynthesis of core-3–derived *O*-glycans showed increased permeability and susceptibility to experimental colitis and cancer (21–23). In contrast to the extensive *O*-glycosylation of the central regions of mucins, component *N*-glycans are in relatively low abundance and largely confined to the end regions of the molecule but are important for mucin dimer formation (24, 25). Because of their high degree of glycosylation (up to 80% of the mucin mass is carbohydrate), mucins constitute potential binding partners for mammalian lectins.

Carbohydrate structures in mucins can trigger DC-mediated immune responses through interactions with members of the C-type lectin receptor (CLR) family (26, 27). Of particular interest are DC-associated C-type lectin (dectin)-1 and -2, which possess single carbohydrate recognition domains (CRDs) in their extracellular region (28, 29). They are expressed mainly in DCs and macrophages. These CLRs have traditionally been associated with the recognition of fungi and bacteria (30), but recent discoveries have revealed emerging roles in homeostasis, autoimmunity, allergy, and recognition of dead and cancerous cells (31). It was recently shown that glycans associated with Muc2 generate gut homeostasis and tolerance by assembling a signal-transducing Dectin-1-FcγRIIB complex on DCs with the help of soluble galectin (Gal)-3 (32). This association resulted in the activation of β-catenin transcription factor that interfered with DC expression of inflammatory, but not anti-inflammatory, cytokines by inhibiting gene transcription through NF-κB (32). Another well-characterized C-type lectin is dendritic cell-specific intercellular adhesion molecule-3-grabbing non-integrin (DC-SIGN), which binds terminal fucose (33). Recently, the lectin domain of DC-SIGN was found to bind to the glycoprotein MUC1 from human milk, preventing pathogen interaction through the presence of Lewis X-type oligosaccharides (34).

Gal-3 is expressed in several cell types and is involved in a broad range of physiologic and pathologic processes, such as cell adhesion, cell activation, chemoattraction, cell cycle, apoptosis, and cell growth and differentiation (35). Earlier studies reported Gal-3 interaction with different mucins, including the ocular cell surface MUC, MUC1 and -16, in a galactose-dependent manner (36) or to cancer mucins from human colon cancer cell lines (37). Structural studies have shown that Gal-3 binds to the TF antigen, Galβ1-3GalNAcα1-Ser/Thr, the mucin core-1 structure of *O*-glycans appearing on the cancer-associated transmembrane mucin protein MUC1 (38, 39).

These observations demonstrate novel ways in which mucins contribute to the regulation of the host immune system. In this study, we showed, using a combination of *in vitro*, cell-based, and force spectroscopy assays that mucins could directly interact with Gal-3, mDectin-2, and SIGN-R1 (the murine homolog of DC-SIGN), providing a structural basis for how mucus delivers tolerogenic signals. Furthermore, we showed that Gal-3 recognized mDectin-1, mDectin-2, and SIGN-R1 *via* carbohydrate-lectin interactions with glycans decorating C-type lectins, uncovering a novel mechanism by which mammalian lectins may modulate inflammatory responses.

## MATERIALS AND METHODS

### Materials

All chemical reagents are from MilliporeSigma (Göttingen, Germany), unless stated otherwise. Recombinant proteins including murine (m)Dectin-1 (mDectin-1) and -2, human (h)Dectin-1 and -2 and DC-SIGN were purchased from R&D Systems (Abingdon, United Kingdom), murine SIGN-R1 was from Sino Biologic (Beijing, China). The NSO cell line was used for mDectin-1 and -1 and hDectin-1 and -2 recombinant production, CHO cell line was used for recombinant production DC-SIGN and human embryonic kidney cells for recombinant SIGN-R1. Antibodies used in the study include mouse monoclonal anti-Gal-3 (R&D Systems) and goat anti-mouse IgG horseradish peroxidase conjugate (MilliporeSigma). The following fluorescein labeled plant lectins, wheat germ agglutinin (WGA), *Ricinus communis* agglutinin (RCA), and *Ulex europaeus* agglutinin (UEA) , were obtained from Vector Laboratories (Peterborough, United Kingdom), β-1,6-galactobiose (galactobiose) from Dextra Labs (Reading, United Kingdom), and scleroglucan from Elicity (Crolles, France). *C3GnT^−/−^* mice were from Dr. Lijun Xia (University of Oklahoma Health Sciences Center, Oklahoma City, OK, USA).

### Cell culture

LS174T cells were cultured in DMEM supplemented with 10% fetal bovine serum, 2 mM l-glutamine, and 1% nonessential amino acids. BWZ.36 reporter cells were cultured in RPMI 1640 media supplemented with 10% fetal bovine serum, 100 U/ml penicillin and streptomycin, 2 mM l-glutamine, and 50 µM 2-ME (R10 medium).

### Gal-3 expression and purification

The cDNA coding for human Gal-3 was synthesized by GenScript (Piscataway, NJ, USA) and supplied in vector pUC57. 5′ Terminal *Nde*I and 3′ terminal *Bam*HI restriction sites were included for subsequent cloning. The Gal-3 encoding cDNA was cloned as an *Nde*I–*Bam*HI fragment into expression vector pET-15b (Novagen, Watford, United Kingdom) which has a thrombin-cleavable N-terminal His6tag and transformed into *Escherichia coli* DH5α to produce plasmid DNA for sequencing. The pET-15b vector with the correct insert sequence was then transformed into *E. coli* expression strain TUNER(DE3) (Novagen). Cultures were grown in lysogeny broth containing 100 µg/ml carbenicillin and 1% (w/v) glucose to OD ∼1.0 at which time protein expression was induced with 1 mM isopropyl β-d-1-thiogalactopyranoside for 4 h at 37°C. Cells were harvested by centrifugation at 10,500 *g* for 10 min at 4°C, resuspended in PBS, and lysed by sonication on ice. Cellular debris was removed by centrifugation at 10,000 *g* for 10 min. The cell lysate containing the recombinant Gal-3 protein was then purified using an α-lactose agarose affinity column (MilliporeSigma). Gal-3 was eluted using 150 mM lactose (MilliporeSigma) in PBS, and all positive fractions for Gal-3 were pooled and buffer exchanged in His-binding solution [20 mM Tris, 500 mM NaCl, 10 mM imidazole (pH 7.9)] using a P10 disposable desalting columns (GE Healthcare, Little Chalfont, United Kingdom). Gal-3-rich fractions were then further purified using immobilized metal ion affinity chromatography and eluted with 250 mM imidazole. Eluted proteins were buffer exchanged with PBS using Vivaspin 6 (3 kDa; MilliporeSigma) spin filter exchange columns. Protein purity was assessed by SDS-PAGE. Purified protein was aliquoted and stored at −20°C.

### ELISA-type assay of binding of Gal-3 to mucins

Native and modified mucins were adsorbed onto a 96-well plate (Maxisorp; Thermo Fisher Scientific, Waltham, MA, USA) at 10 µg/ml in PBS overnight at 4°C. Wells were washed with PBS containing 0.05% Tween-20 and blocked with 1% bovine serum albumin in PBS for 2 h. Wells were sequentially washed and incubated with 5 µg/ml of Gal-3 (diluted in blocking buffer) for 2 h at 37°C. For the inhibition assays, Gal-3 was prepared in blocking buffer containing 100 mM lactose. After further washes, a mouse monoclonal anti-Gal-3 was added at a 1:5000 dilution in blocking buffer and incubated at 37°C for 1 h. Wells were washed as above and goat anti-mouse IgG horseradish peroxidase conjugate was added to each well at a 1:5000 dilution and incubated for 45 min at 37°C. After final washes, the presence of Gal-3 was detected by addition of TMB High Sensitivity Substrate Solution (BioLegend, San Diego, CA, USA). The colorimetric reaction was stopped by adding an equal volume of 2 N sulfuric acid (H_2_SO_4_) and read at 450 nm with a Benchmark Plus Microtiter Plate Reader (Bio-Rad, Hercules, CA, USA).

### Atomic force microscopy and force spectroscopy

The atomic force microscope used in this study was an MFP-3D BIO (Asylum Research, Santa Barbara, CA, USA). Force spectroscopy experiments were conducted by using DC mode in Dulbecco’s PBS (MilliporeSigma).

#### Tip and slide functionalization

Silicon nitride atomic force microscopy (AFM) probe tips (PNP-TR; NanoWorld, Neuchâtel, Switzerland) were functionalized by using a 4-step procedure (at 21°C). In the first step, the tips were incubated in a 2% solution of 3-mercaptopropyltrimethoxy silane (MilliporeSigma) in dried toluene (4 Å molecular sieve) for 2 h, followed by washing with toluene and then chloroform. In the second step, the silanized tips were incubated for 1 h in a 0.1% solution of a linear heterobifunctional polyethylene glycol (PEG) linker in chloroform, which contains a maleimide (MAL)and a succinimidyl *N*-hydroxysuccinimide ester group [2 kDa MAL-PEG- succinimidyl carboxyl methyl ester (SCM); Creative PEGWorks, Durham, NC, USA], on each end of the PEG chain. MAL reacts with the mercapto groups (SH) on the AFM tip and SCM reacts with primary amines in the receptor proteins. Unbound MAL-PEG-SCM was washed off with chloroform, and the tips were dried with argon. The third step involved covalent attachment of lectins (Gal-3, mDectin-1, mDectin-2, SIGN-R1, hDectin-1, hDectin2, and DC-SIGN) by incubation of the tips in 1 mg/ml lectin solutions in PBS (pH 7.4) for 1 h at 21°C, followed by a PBS washing step. In the fourth step, the lectin-functionalized cantilevers were incubated in a 10 mg/ml solution of glycine in PBS to amine cap any unreacted succinimide groups, followed by washing in PBS. Lectin-functionalized tips were stored under PBS at 4°C overnight before use.

#### Attachment of receptors onto glass slides

The functionalization of glass slides was performed by initial silanization with 3-mercaptopropyltrimethoxy silane, as for the AFM tips, followed by attachment of a different heterobifunctional linker molecule, *N*-γ-maleimidobutyryl-oxysuccinimide ester (Thermo Fisher Scientific). A drop (100 μl) of the receptor molecules (mDectin-1, Gal-3, or mucins) at 1 mg/ml in PBS was placed onto the derivatized slides for 1 h at room temperature to link the proteins *via* their N termini, followed by washing in PBS. The surfaces were incubated in a 10 mg/ml solution of glycine to amine cap any unreacted succinimide groups on the glass, and then washed in PBS to remove any unbound glycine, before being inserted into the liquid cell of the AFM.

#### Enzymatic treatment on slide

The functionalized slides were transferred into a sealed glass box with a damp section of tissue on the base to prevent evaporation during the enzymatic treatment. Peptide *N*-glycosidase (PNGase) F (200 µl; MilliporeSigma) at 100 U/ml in 50 mM ammonium bicarbonate (pH 8.4), were added to the functionalized sections of the slides and incubated for 12 h at 37°C_._ The slides were then rinsed with 12 × 200 µl of PBS before analysis by force spectroscopy.

#### Force spectroscopy measurements

All binding measurements to the potential receptor molecule-coated glass surfaces were performed in PBS. For inhibition studies, relevant cognate sugars (lactose, yeast α-mannan, or scleroglucan) in PBS were added into the liquid cell at various concentrations. The experimental data were captured in a so-called force–volume mode (at a rate of 2 μm/s in the *Z* direction, at a scan rate of 1 Hz, and at a pixel density of 32 × 32). In this mode, the instrument ramps the *Z* piezo element of the scanner by a predetermined amount at each sample point over a selected scan area (3 μm) and records the subsequent deflection of the cantilever as it is pushed toward (maximum load force 300 pN), then retracted from the sample surface. This produces a matrix of 1024 force *vs.* distance curves for each tip–sample combination. The spring constant *k* of the cantilevers was determined by fitting the thermal noise spectra (40), yielding typical values in the range of 0.01–0.04 N/m.

#### Analysis of force–distance curves

The adhesion force maps (labeled in a cold–warm color gradient) were calculated by measuring the difference between the minimum values seen in the retraction force curves and the final 10 points where the AFM tip is sufficiently distant from the sample surface so that the cantilever has no deflection and the force is 0. Therefore, this region of the force curve is linear and defined as the baseline (MFP-3D software v.111111+1610; Asylum Research).

Force *vs.* distance data were also analyzed by using a custom-made Excel (Microsoft, Redmond, WA, USA) macro (41). This approach ensured that any nonspecific tip–sample interactions, which appear at the tip–glass detachment point, were eliminated from the measurements. The distance between adhesive events and the tip–sample detachment point (0 nm in the *x* axis) in the force spectra were also determined by using the macro. Peak identification in the retraction data was performed by identifying turning points, and discrimination from noise was achieved with a user-adjustable value, which set the threshold level at a 6× multiple of the noise level amplitude.

### Reporter cell assays

BWZ.36 cells expressing murine CLRs mDectin-1, mDectin-2, and SIGN-R1 were established (42, 43). In brief, the extracellular domain of mDectin-1 (Ser74 through Leu244), mDectin-2 (Gln49 through Leu209), or SIGN-R1 (Ser76 through Gly324) was cloned into the retrovirus vector pMXs-IRES-EGFP-Ly49A-CD3 harboring the transmembrane region of the mouse Ly49A and the cytoplasmic domain of the mouse CD3ζ (42). The resulting pMXs vectors encoding mDectin-1, mDectin-2, and SIGN-R1 were used for the retrovirus transduction using Plat-E cells (42). To introduce 2 amino acid mutations into the carbohydrate-binding domain of mDectin-2 (E168Q and N170D) and SIGN-R1 (E285Q and D287N), DNA fragment encoding extracellular domain of mDectin-2 and SIGN-R1 with the 2 missense mutations was synthesized (GenScript) and used for cloning as above.

To assess the binding of different ligands to the C-type lectin domain, specific ligands were adsorbed on a 96-well microplate (Thermo Fisher Scientific) overnight at 4°C in PBS. Wells were washed twice with PBS and BWZ.36 cells (expressing C-type lectins or mock cells) were added to the wells at 5 × 10^5^ cells/ml and incubated for 18 h at 37°C and 5% CO_2_. After incubation, cells were centrifuged at 510 *g* for 3 min and the supernatant discarded. To measure β-galactosidase activity encoded by the *lacZ* reporter gene, 200 µl of 150 mM chlorophenol red-β-D-galactopyranoside diluted in a CPRG assay reaction buffer (PBS supplemented with 0.125% Triton X-100, and 100 mM 2-ME) was added to each well. The plate was incubated for 45 min at 37°C, 5% CO_2_ before measurement of color development (A570/630 nm) with a Benchmark Plus Microtiter Plate Reader (Bio-Rad).

### Mucin extraction

Mucin extraction was performed as described in Davies *et al*. (44). Culture medium from the LS174T cell line was freeze dried before extraction of MUC2. After freeze drying, samples were solubilized overnight in a 5 M guanidine chloride (GuCl) buffer containing protease inhibitors (7.95 mM EDTA, 12.25 mM benzamidine, 6.25 mM *N*-ethylmaleimide, 1.25 mM PMSF, 3.75 mM sodium azide, 0.1 mg/ml soy bean inhibitor). Samples were centrifuged at 12,000 rpm. The pellet was reduced with 10 mM DTT for 4 h at 45°C and alkylated with 25 mM iodoacetamide overnight before dialysis against 50 mM ammonium bicarbonate. The same protocol was followed for the mucus scraped from SI and colon of wild-type (WT) C57BL/6 and *C3GnT^−/−^* mice (21). Access to mouse tissues was performed under the Animal Welfare and Ethical Review Body of University of East Anglia’s establishment license (according to Home Office requirements). Porcine gastric mucin (PGM) type III; MilliporeSigma) was further purified with purified porcine gastric mucin (pPGM) (45). The purity of the mucins was determined by immunoblot analysis, lectin, and Alcian blue staining after electrophoresis by SDS-PAGE or vertical agarose gel electrophoresis (46).

### Characterization of purified mucins

Mucin glycoproteins were separated by vertical SDS‐agarose gel electrophoresis (46). In brief, a 1 cm 12% polyacrylamide plug was cast using 10 × 8 cm glass plates and 1.5 mm spacers (Bio‐Rad). After polymerization, the preparation was overlaid by a 1% agarose gel (Agarose LE; Melford Biolaboratories, Ipswich, United Kingdom) prepared in running buffer (50 mM Tris, 384 mM glycine, and 0.1% SDS) containing 30% glycerol. Samples were electrophoresed at 15 mA for 65 min at 4°C in a Mini‐Protean Tetra Cell device (Bio‐Rad). For electroblotting, gels were placed in NuPAGE transfer buffer (Thermo Fisher Scientific) containing 10% methanol and blotted to 0.45 μm Hybond PVDF membrane (Amersham/GE Healthcare Life Sciences, Little Chalfont, United Kingdom) at 40 V for 2 h 20 min at 4°C in an Xcell II blot module (Thermo Fisher Scientific). To assess for the presence of smaller proteins, extracted mucins were separated by SDS-PAGE on a 4–12% Bis-Tris NuPAGE gel (Thermo Fisher Scientific) in 3-(*N*-morpholino) propane sulfonic acid–SDS running buffer according the manufacturer’s protocol. The gel was stained with Coomassie blue using Instant Blue stain protein solution (Expedeon; MilliporeSigma). The proteins were transferred onto a 0.45 μm pore size Hybond PVDF membrane (Amersham/GE Healthcare Life Sciences).

For immunolabeling or lectin staining, the membrane was first blocked with Protein-Free blocking buffer (Thermo Fisher Scientific) for 60 min and incubated with FITC-WGA lectin at 2.5 μg/ml for 1 h at room temperature or with mouse anti‐MUC2 (Abcam, Cambridge, United Kingdom) diluted 1:1000 in the blocking buffer overnight at 4°C. The blots were washed with PBS and 0.1% Tween 20. For immunodetection, the blots were incubated with AlexaFluor 488-conjugated goat anti‐mouse IgG (Thermo Fisher Scientific) diluted 1:2000 for 1 h in the blocking buffer, and rinsed with PBS-0.1% and Tween-20. The blots were imaged with a Pharos FX Plus Imager (Bio-Rad).

For Alcian blue staining, the membrane was rinsed with pure H_2_O and transferred into an Alcian Blue solution (25% (v/v) ethanol, 10% (v/v) acetic acid, and 0.125% (w/v) Alcian Blue *8GX* for 30 min. The blots were destained with 100% methanol for 15 min, rinsed with pure H_2_O, air dried, and imaged with a GS-800 calibrated densitometer (Bio-Rad).

### Enzymatic treatment

For *N*-deglycosylation of mucins by PNGase F, 1 mg of colon Muc2 from WT and *C3GnT^−/−^* mice was treated with 10 U PNGase F (Millipore Sigma) for 24 h at 37°C in 50 mM ammonium bicarbonate buffer. The samples were then centrifuged at 15,000 *g* for 5 min. The pellets were washed 3 times with water, and the resulting PNGase F-treated mucins used for binding assays.

### Mass spectrometry–glycosylation analysis

#### Release of oligosaccharides from mucin by alkaline borohydride treatment

The mucins were subjected to β-elimination under reductive conditions (0.1 M NaOH and 1 M NaBH4) for 20 h at 45°C. The reaction was stopped by adding Dowex 50×8 (Dow Chemical Company, Midland, MI, USA) and filtered before being coevaporated with methanol 3 times. The remaining salts were removed by Carbograph (Grace Alltech, Columbia, MD, USA).

#### *Permethylation of* O*-glycans*

Permethylation was performed on released *O*-glycans from the different mucin samples. Samples were solubilized in 200 μl DMSO, followed by addition of ∼25 mg NaOH and 300 μl iodomethane under anhydrous conditions and vigorously shaken at room temperature for 90 min. The permethylation reaction was stopped by addition of 1 ml acetic acid (5% v/v). Permethylated *O*-glycans were purified on a hydrophilic–lipophilic balanced copolymer Oasis cartridge (Waters, Hertfordshire, United Kingdom). In brief, cartridges were activated by 100% methanol, equilibrated with methanol:water (5:95, v/v), and the samples loaded onto the cartridges. The cartridges were washed with methanol:water (5:95, v/v) and the permethylated *O*-glycans were eluted with methanol.

#### *Analysis of permethylated* O*-glycans by mass spectrometry*

Matrix-assisted laser desorption and ionization–time of flight mass spectrometry (MALDI-TOF MS) and TOF/TOF-MS data were acquired with the Autoflex analyzer mass spectrometer (Bruker, Coventry, United Kingdom) in the positive-ion and reflectron mode. Samples were dissolved in methanol/water (1:1 v/v) and mixed with an equal volume of 2,5-dihydroxibenzoic acid (10 mg/ml in 70:30 methanol:water; MilliporeSigma) and spotted onto an MTP 384 target plate polished steel TF (Brucker). Six spectra of 500 shots were accumulated from a range of *m*/*z* 500 to 4000. Peaks observed in the MS spectra were selected for further tandem MS (MS/MS) analysis without the collision-induced dissociation mode. MS/MS data comprises a total of 50 subspectra of 2000 shots.

### C-type lectin glycosylation analysis

Each C-type lectin (50 μg mDectin-1, mDectin-2, and SIGN-R1) was incubated in 50 mM ammonium bicarbonate buffer with 10 U PNGase F (recombinant PNGase F of *Flavobacterium meningosepticum*; MilliporeSigma) for 24 h at 37°C. The reaction was stopped by freeze drying. The released *N*-glycans were then purified on C18-SepPak cartridges (Waters). Cartridges were activated by 5 ml methanol, equilibrated with 5 ml acetic acid (5%, v/v), 5 ml acetonitrile, and 10 ml of acetic acid (5%, v/v). Samples were solubilized into 200 μl acetic acid (5%, v/v) and loaded onto the cartridges. The flow-through was collected, and cartridges were washed with 10 ml acetic acid (5%, v/v) and the solutions freeze dried before the permethylation of purified *N*-glycans.

### *N*-glycosylation analysis of mucins

Colon Muc2 (1.5 mg) from WT and *C3GnT^−/−^* mice was digested by 500 μg of trypsin in 50 mM ammonium bicarbonate buffer at 37°C for 24 h. Trypsin was inactivated by heating the samples for 5 min at 100°C. The samples were freeze dried, and the purification of mucin peptides was performed with a hydrophilic–lipophilic balanced Oasis cartridge (Waters). The cartridges were activated and equilibrated with 5 ml methanol, 5 ml acetic acid (5% v/v), 5 ml propan-1-ol, and 15 ml acetic acid (5% v/v). The digested samples were loaded onto the cartridges, washed with 20 ml acetic acid (5% v/v), and eluted with 20% (v/v) and 40% (v/v) of propan-1-ol. The eluate was evaporated under nitrogen before freeze drying.

The eluted peptides were dissolved in 50 mM ammonium bicarbonate and treated with 10 U PNGase F at 37°C for 24 h. Released glycans were purified on a Sep-Pak C18 Classic cartridge (Waters), prewet with 5 ml methanol and equilibrated with 5 ml acetic acid (5% v/v), 5 ml acetonitrile, and 10 ml acetic acid (5% v/v). After the samples were loaded onto the cartridge, the released glycans were eluted with 10 ml acetic acid (5% v/v). The eluant was freeze dried and prepared for MALDI-TOF-MS analysis.

### Permethylation of *N*-glycans

Permethylation was performed on released *N*-glycans from the different C-lectins and mucins. Samples were solubilized in 200 μl DMSO, and 25 mg NaOH (1.25 M in final concentration) and 300 μl iodomethane were added in anhydrous conditions followed by vigorous shaking at room temperature for 90 min. The permethylation reaction was stopped by addition of 1 ml acetic acid (5% v/v). Permethylated *N*-glycans were extracted by chloroform. The chloroform phases were washed 3 times with acetic acid (5% v/v) and 3 times with water before evaporation under nitrogen. The permethylated *N*-glycans were then purified on C18-SepPak cartridges. Cartridges were conditioned sequentially with 5 ml methanol, 5 ml water, 5 ml acetonitrile, and 10 ml water. Permethylated *N*-glycans were resuspended into 200 μl methanol:water (50:50 v/v), loaded onto a C18-SepPak cartridge, washed with 15 ml water and 2 ml acetonitrile (10% v/v), and eluted sequentially with 1 ml (35% v/v), 1 ml (50% v/v), and 2 ml (80% v/v) acetonitrile. The eluate was evaporated under nitrogen before freeze drying and was analyzed by MS with the Bruker Autoflex Analyzer Mass Spectrometer (Thermo Fisher Scientific) in the positive-ion and reflectron mode by using 2,5-dihydroxibenzoic acid (10 mg/ml in 70:30 methanol:water; MilliporeSigma) as the matrix.

### Mucin chemical deglycosylation

#### Desialylation of mucins

Mucins were desialylated by 0.1 M trifluoroacetic acid (TFA; MilliporeSigma) treatment at 80°C for 1 h and then dialyzed against water.

#### *Sequential de-*O*-glycosylation of mucins*

After desialylation, de-*O*-glycosylation based on the oxidation and elimination method was performed according to published methods (47). In brief, 2.5 μg of mucins were coated onto a 96-well microplate (Fluotrac/Greiner Bio-One; MilliporeSigma). Mucins were incubated with 200 μl 10 mM sodium metaperiodate in 0.1 M sodium acetate buffer at 37°C for 1 h. The wells were washed 3 times with H_2_O and incubated with 25% ammonium hydroxide at room temperature for 1 h. Three cycles were performed to sequentially remove monosaccharides from mucin glycans. Finally, the samples were treated with 25% NH_4_OH at 37°C for 2 h after the last periodate oxidation (37°C for 1 h) to remove any core-GalNAc.

To test the efficiency of deglycosylation assays, native and chemically treated mucins were tested against a range of FITC-conjugated lectins, including WGA, RCA, and UEA. After treatment, mucins were washed with PBS and blocked overnight with Pierce Protein-Free Blocking Buffer (Thermo Fisher Scientific). The wells were washed with PBS and 0.05% and Tween-20 and incubated with FITC-labeled lectins (dilution 1:1000 in blocking buffer) for 1 h in the dark. After a final wash with PBS, the fluorescence was detected by excitation at 488 nm and emission at 525 nm using a FluoStar Optima Microplate Reader (BMG Labtech, Aylesbury, United Kingdom). Confirmation was also obtained by MS analysis with deglycosylated purified pPGM.

### Statistical analysis

A 1-way ANOVA, followed by Tukey’s test, or 2-way ANOVA were used for statistical analysis with Prism software (GraphPad Software, La Jolla, CA, USA). A value of *P* < 0.05 was considered significant.

## RESULTS AND DISCUSSION

### Gal-3 binds to mucins *via* poly(LacNAc) glycans

Galectins represent a family of carbohydrate-binding proteins with affinity for β-galactoside sugars. Gal-3, the only chimera galectin found in vertebrates, consists of 1 CRD connected to a collagen-like sequence formed by Pro-Gly-Tyr tandem repeats and an N-terminal domain (35). Gal-3 can accommodate various β-galactosides in its canonical-binding site, with high affinity toward histo-blood group epitopes, ganglioside GM1-pentasaccharide and clustered core-1 *O*-glycans. Gal-3 recognizes *N*-acetyllactosamine (LacNAc; Galβ1-4GlcNAc), and, in particular, internal LacNAc within poly-*N*-acetyllactosamine (poly-LacNAc) (48, 49). Gal-3 has been shown to interact with glycosylated MUC2 *via* the formation of a complex with Dectin-1 and FcγRIIB on DCs enhancing gut homeostasis and oral tolerance (32). However, the nature of *O*-glycans mediating the interaction of Gal-3 with mucins forming the intestinal mucus layer in the GI tract has not been investigated.

**Figure 1.**
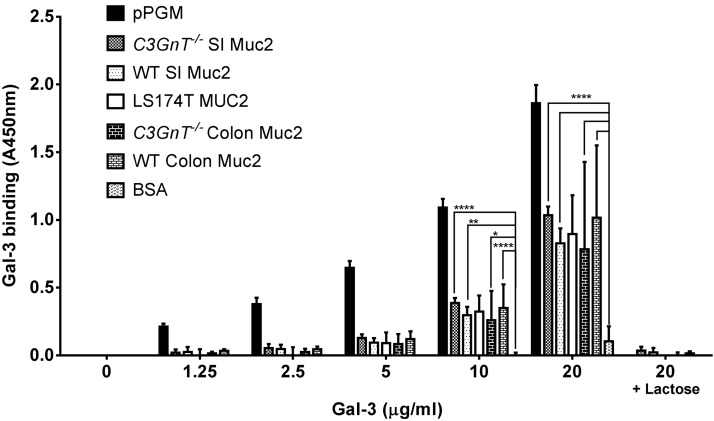
Binding of Gal-3 to mucins is dose dependent and inhibited by lactose. Recombinant Gal-3 was incubated at different concentrations (0, 1.25, 2.5, 5, 10, and 20 μg/ml) for 2 h at 37°C on a 96-well plate coated with 100 μl of mucins (10 μg/ml). The binding was assessed against MUC6 (pPGM), Muc2 from WT or *C3GnT^−/−^* mice purified from SI or colon (C), MUC2 purified from LS174T cells, and bovine serum albumin (negative control). The binding of Gal-3 was inhibited against all the mucins coated at 20 μg/ml in the presence of 100 mM lactose solution. Data are representative of 3 independent experiments with similar results. Error bars, denote the sd. Statistical significance of differences were determined by 2-way ANOVA. *P < 0.05, **P < 0.01, ****P < 0.0001.

We showed that Gal-3 binds to a range of mucins purified from different sources including gel-forming MUC2 from human cell lines LS174T, Muc2 from SI and colon from murine models, conventionally raised WT C57BL/6 mice or *C3GnT^−/−^* mice (21), and MUC6 purified from commercial pPGM (Supplemental Figs. S1 and S2). Binding was stronger to pPGM as compared to mouse Muc2 and MUC2 from LS174T cells (**Fig. 1**). Significant binding to Gal-3 was observed for all mucins tested at 20 μg/ml, and binding was completely inhibited by 100 mM lactose, suggesting that binding occurs through Gal-3 CRD. Gal-3 bound to pPGM in a concentration-dependent manner. No significant differences were found within the murine mucins (WT and *C3GnT^−/−^*) purified either from the SI or colon. Higher variability in Gal-3 binding to Muc2 from WT and *C3GnT^−/−^* mouse colon may be related to lower solubility of colonic Muc2 at higher concentration.

**Figure 2.**
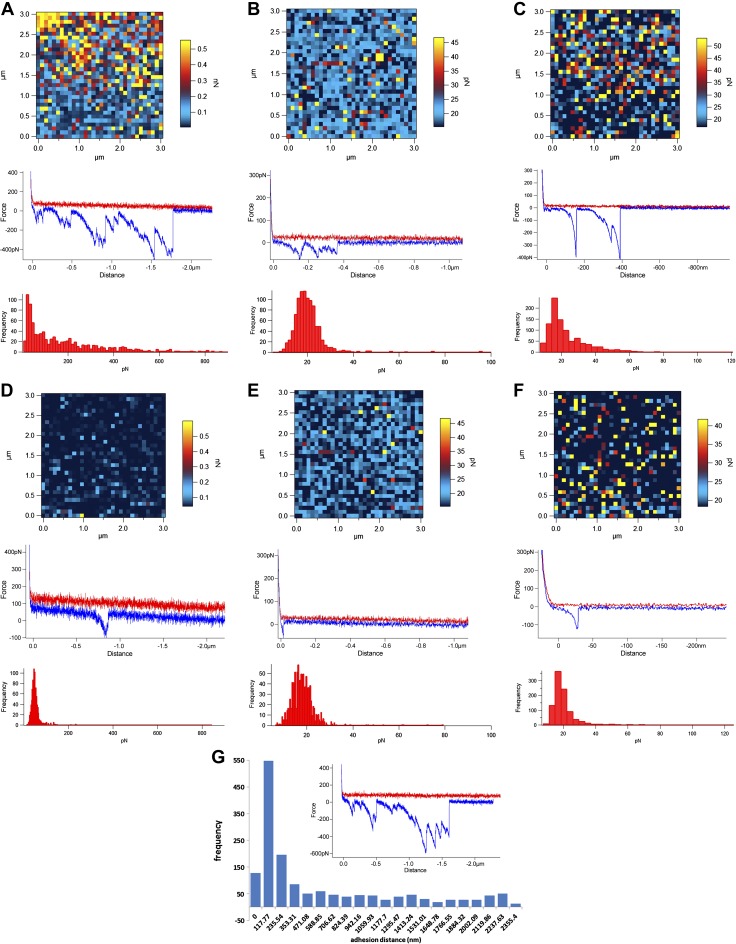
Force spectroscopy of Gal-3 interactions with mucins. Adhesion maps, examples of force curves, and adhesion histograms of Gal-3 with MUC6 pPGM in PBS (*A*) or in the presence of 200 mM lactose (*D*); colonic Muc2 from *C3GnT^−/−^* mice in PBS (*B*) or in the presence of 200 mM lactose (*E*); MUC2 from LS174T cells in PBS (*C*) or in the presence of 200 mM lactose (*F*). *G*) Gal-3–pPGM adhesion separation distances. Inset: example of a force–distance curve. Gal-3 was attached to the AFM tip *via* a PEG linker, and mucins were covalently attached to the glass slide *via* their N termini, resulting in the mucin glycan chains protruding into the aqueous liquid buffer.

The interaction between Gal-3 and the different mucins was further investigated by direct single molecular measurement with force spectroscopy (**Fig. 2**). Mucins were covalently attached to the glass surface *via* their N-termini, enabling the mucin glycan chains to protrude into the aqueous liquid buffer, and Gal-3 was covalently attached to the AFM tip. Figure 2 includes adhesion force maps (top panels) revealing the level of adhesive events in the force–distance–scanned regions, example force–distance curves (middle panels), and histograms (bottom panels) that show the quantification of the adhesive events detected across the entire 1024 force spectra within the force volume–mapped regions. In the example, force–distance curves, the red line represents the ligand functionalized tip approaching the sample surface from several micrometers and the blue line the retraction force data collected after the tip engaged with the sample. When adhesion occurs, negative peaks appear in the retraction curves (but not in the approach curves). The negative peaks are nonlinear, because both the PEG linker, on the functionalized tip, and the mucin chains attached to the glass slide are stretched until the ligand–receptors are pulled apart. The example force–distance curves between Gal-3 and the different mucins tested showed specific interactions occurring at distances significantly away from the tip–sample detachment point, reflecting the distribution of glycan chains along the mucin molecules (Fig. 2*A*–*C*). Gal-3 binding was stronger to MUC6 pPGM (Fig. 2*A*) than to mouse colonic Muc2 (Fig. 2*B*) or MUC2 from LS174T cells (Fig. 2*C*), in agreement with the ELISA results. The addition of free lactose led to a reduction in the frequency of the interactions (Fig. 2*D*–*F*), confirming that Gal-3 bound to mucins *via* its CRD, as previously reported for pPGM (50). Furthermore, the Gal-3 adhesion separation distances spread between 117 and 2355 nm with the inset example force curve showing higher multiple adhesion events over the range of the contour length of mucin chains (Fig. 2*G*), as compared to Gunning, *et al*. (50).

**Figure 3.**
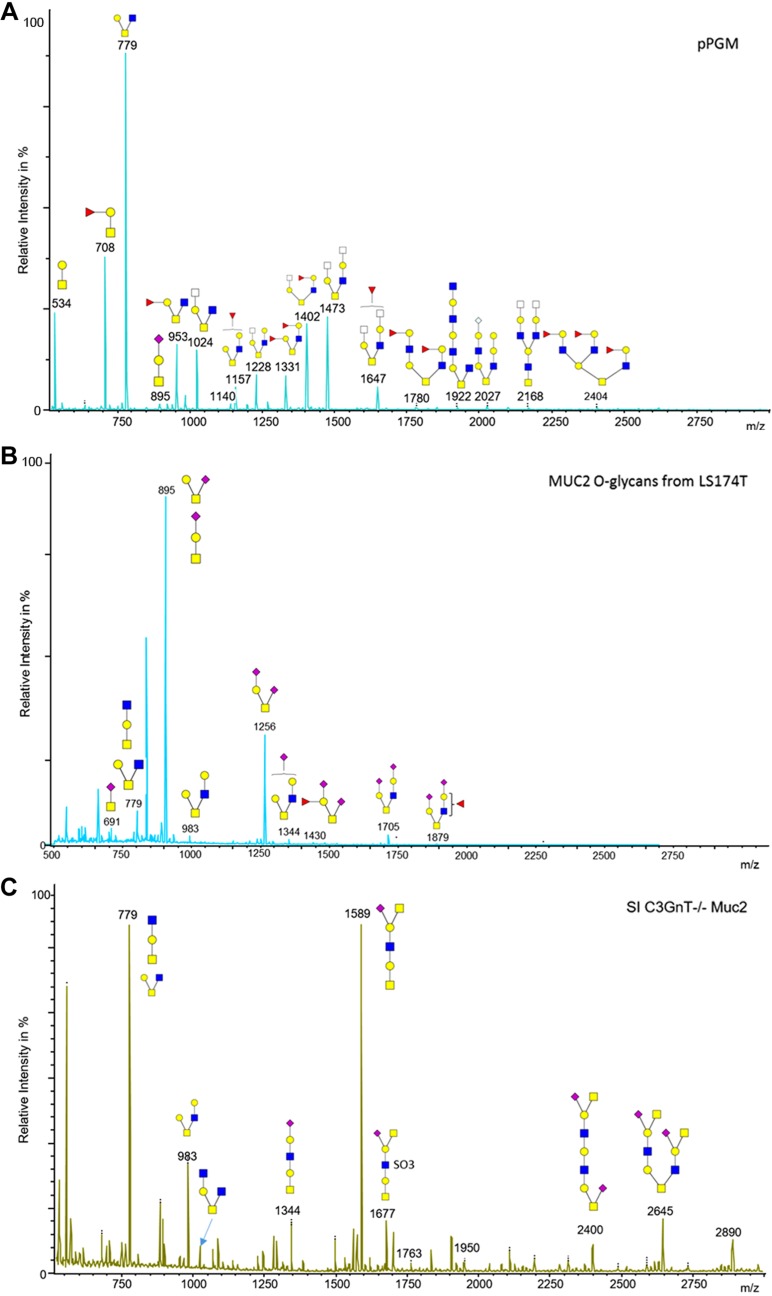

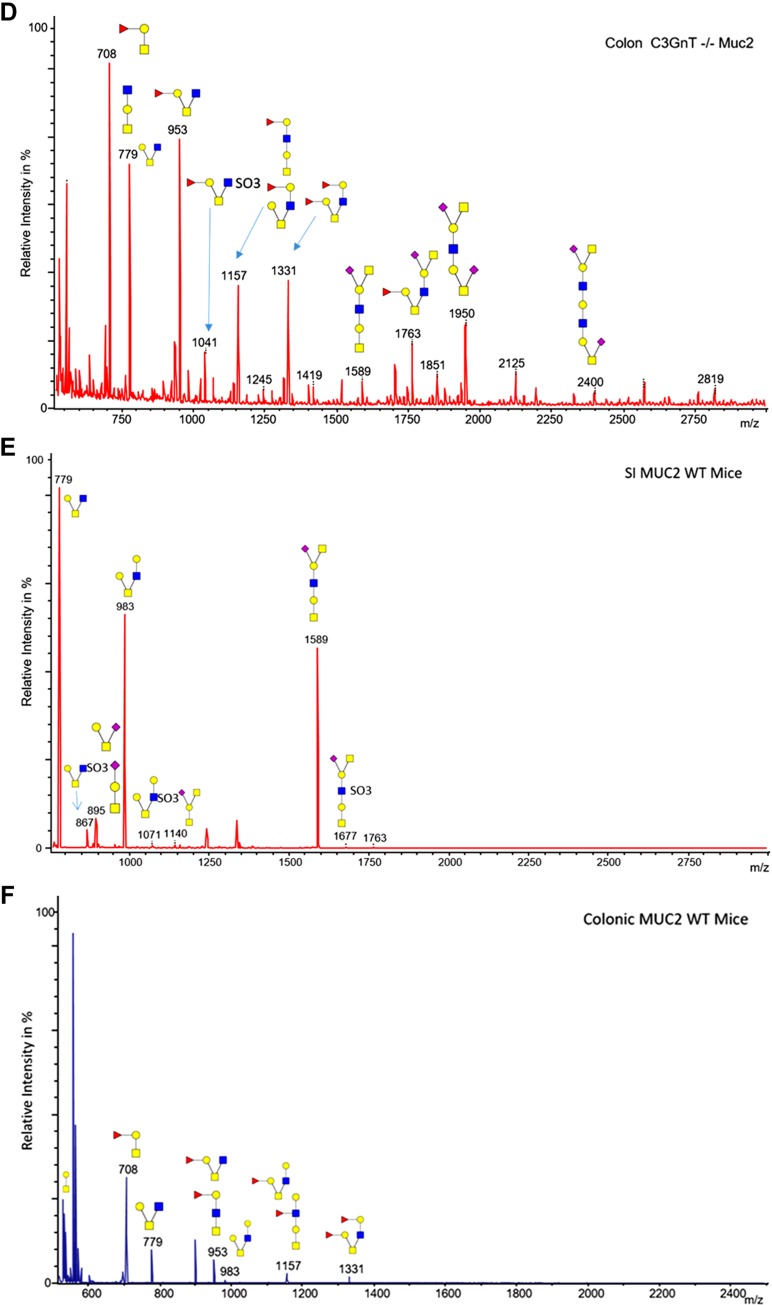
Structural characterization of mucin *O*-glycans by MS. MALDI-MS spectra, acquired in the positive-ion mode [M + Na]^+^ of permethylated *O*-glycans from pPGM (*A*), MUC2 from LS174T cells (*B*), Muc2 from SI (*C*) and colon (*D*) of *C3GnT^−/−^* mice, and Muc2 from SI (*E*) and colon (*F*) of WT mice. Monosaccharide symbols follow the Symbol Nomenclature for Glycans system (81). Key: fucose (red triangle), GlcNAc (blue square), sialic acid (purple diamond), galactose (yellow circle), and HexNAc (off-white square).

**Figure 4.**
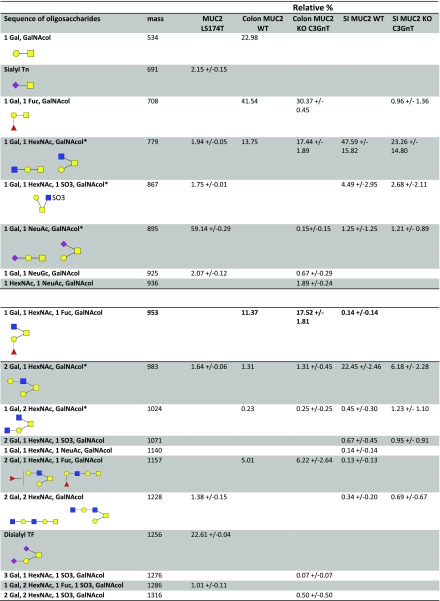

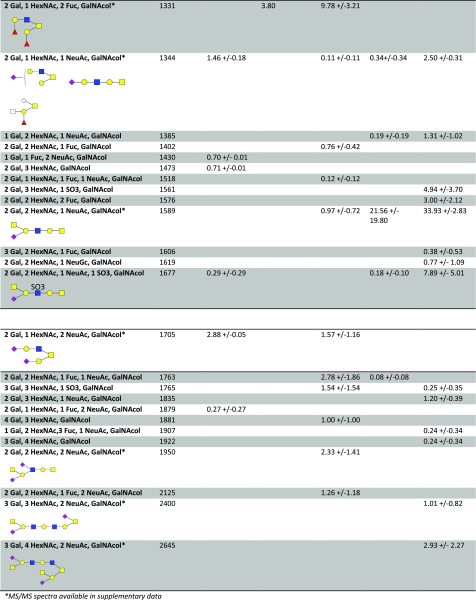
Summary of the mucin *O*-glycan structures as determined by MS. These include LS174T MUC2, colon WT Muc2, colon *C3GnT^−/−^* Muc2, SI WT Muc2, SI *C3GnT^−/−^* Muc2. The relative percentage of each structure is calculated as: [area of the peak/(sum of peaks areas corresponding to *O*-glycans)] × 100.

To gain further insight into Gal-3 binding specificity to mucin glycans, the glycosylation profile of purified mucins was determined by MS. The *O*-linked oligosaccharides were released from reduced mucins by reductive β-elimination, permethylated, and analyzed by MALDI-TOF and TOF/TOF-MS (**Figs. 3** and **4**). pPGM showed core-1, derived-core-1, core-3, and derived-core-3 *O*-glycans, in agreement with another study (51). A total of 41 different *O*-glycans composed of up to 11 monosaccharides were identified, with only 4% of the structures being sialylated. The glycosylation profile of pPGM was dominated by short *O*-glycans up to 6 monosaccharides from *m*/*z* = 534 to *m*/*z* = 1473. These glycans represent ∼60% of the total of pPGM *O*-glycans (Fig. 3*A*). LS174T MUC2 was characterized by the presence of 2 major *O*-glycans, the sialyl-TF at *m*/*z* = 895 and the di-sialyl-TF at *m*/*z* = 1256 (constituting about 70% of the total *O*-glycans identified in LS174T MUC2). The proportion of sialylation was about 92%, in line with previous reports showing that mucins from LS174T were highly sialylated (52, 53). No large structures with poly-LacNAc were observed (Fig. 3*B*). In *C3GnT^−/−^* SI Muc2, 58% of *O*-glycans were sialylated, and only 5% were fucosylated (Fig. 3*C*), whereas in colonic Muc2, 34% of *O*-glycans were sialylated and 64% were fucosylated (Fig. 3*D*). In these mucins, *O*-glycans were core-1 and derived-core-1, in agreement with previous reports (19, 54). *C3GnT^−/−^* mice showed a compete ablation of all core-3–derived *O*-glycans on colonic mucins, but a retention of core-1– and -2–derived *O*-glycans as analyzed by MALDI-TOF MS (21) and confirmed in a recent study (54). Further characterization of *C3GnT^−/−^* mice revealed that loss of core-3–derived *O*-glycans led to a partial exposure of the Tn antigen compared with WT mice (21). In WT murine SI Muc2, we observed sialylated structures with Sda/Cad antigen at *m*/*z* = 1140 and 1589, characterized in MS/MS by the presence of the CY ion at *m*/*z* = 606 (Supplemental Fig. S3) (55) and the presence of sialyl TF at *m*/*z* = 895. The colonic WT mouse Muc2 was characterized by the presence of short fucosylated *O*-glycans with blood group H antigen (Fucα1_2Gal-) characterized in MS/MS by the presence of the C-ion at *m*/*z* = 433 (Supplemental Fig. S3), but no sialylated *O*-glycans were detected in contrast with previous results (54), whereas *C3GnT^−/−^* colonic Muc2 showed the presence of sialylated *O*-glycans at *m*/*z* = 1589, 1950, and 2400. *O*-glycans were predominantly core-1 and -2 structures in agreement with previous results (54).

**Figure 5.**
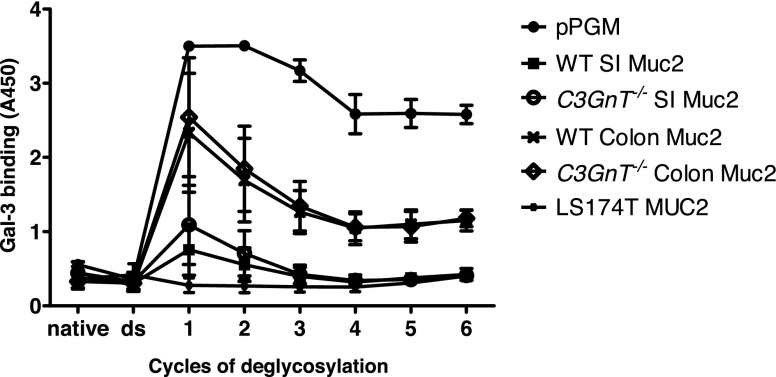
Binding of Gal-3 to mucins is increased after desialylation (ds) and several cycles of deglycosylation. MUC6 (pPGM), Muc2 from WT or *C3GnT^−/−^* mice purified from SI or colon (C), and MUC2 purified from LS174T cells were coated at 10 μg/ml (100 μl/well). Desialylation by mild hydrolysis followed by 6 cycles of deglycosylation were performed directly in the microplate. The binding of Gal-3 to native and modified mucins was assessed after incubation for 2 h at 37°C, followed by colorimetric reaction. Data are representative of 3 independent experiments with similar results. Error bars denote the sd.

These structural analyses suggest that the presence of terminal sialylation and fucosylation in LS174T MUC2 and murine Muc2 hampers recognition of Gal-3 binding epitopes in mucins. To further determine the specificity of Gal-3 to intestinal mucin glycans, immobilized mucins were subjected to cycles of deglycosylation by chemical treatment based on the oxidation or elimination method (47). After a mild acid hydrolysis that removed monosaccharides with weak linkages, such as sialic acid, terminal monosaccharides were removed sequentially by oxidation and elimination, as monitored by ELISA with plant lectins (WGA, UEA, and RCA; data not shown). This process was further confirmed by MS of pPGM (Supplemental Fig. S4 and Table S1). Poly-LacNAc structures were detected after desialylation and after the first cycle of deglycosylation, which removes all fucose residues and terminal monosaccharides. We observed, in particular, an increase in structures at *m*/*z* = 1228, 1473, 1677, 1718, 1922, and 2168. These structures were characterized by a sequence of Gal and GlcNAc which forms at least 2 LacNAc motifs. Binding of Gal-3 to all mucins, except MUC2 from LS174T cells, was significantly augmented after the first cycle of deglycosylation that removed all the fucose residues or terminal sugars (**Fig. 5**). The proportion of these structures decreased after the second cycle of deglycosylation in favor of smaller structures at *m*/*z* = 534, 575, 779, and 1024, which may explain the decrease in interaction with Gal-3. The binding of Gal-3 to MUC2 from LS174T cells did not increase after deglycosylation, which may be related to the presence of short glycan structures. Our data show that Gal-3 preferentially binds to poly-LacNAc structures that become available after the removal of terminal monosaccharides in mucin glycan chains. These data are consistent with results in previous studies showing that Gal-3 binds only glycans containing at least 3 repeating type 2 LacNAc/lactose structures that lacked branched features (56, 57).

### Mucin binds to mDectin-2 and SIGN-R1, but not mDectin-1, *via* lectin–carbohydrate interactions

Glycans are recognized by C-type lectins expressed mostly by myeloid antigen-presenting cells, such as macrophages and DCs. CLRs belong to the family of antigen uptake receptors, as they recognize cell surface glycans on many viruses, bacteria, and parasites, but may bind and respond to host endogenous glycoproteins as well. CLRs recognize a diverse range of neutral sugars, including mannose, fucose, glucose, or β-glucan (27, 58). These CLRs possess a CRD and are capable of mediating intracellular signaling either directly, through intracellular signaling domains, or indirectly, by associating with signaling adaptor molecules. Dectin-1 recognizes β-glucans *via* interaction with its CRD and transduces signals through its immunoreceptor tyrosine-based activation motif in the cytoplasmic domain (29), whereas Dectin-2 recognizes α-mannans and transduces its signal through association with the immunoreceptor tyrosine-based activation motif–containing FcR γ chain (59). SIGN-R1 is the murine homolog of DC-SIGN (60). Ligands for DC-SIGN include fucosylated structures, including blood group antigens, human milk glycans (61, 62) or MUC1 *via* Lewis X structures (34), as well as high-mannose moieties (63, 64). SIGN-R1 was found to be a major mannose receptor for fungal and other pathogens (65). Dectin-2 clearly recognizes mannose-type ligands, although the precise nature of these ligands differs from those recognized by SIGN-R1 (66).

**Figure 6.**
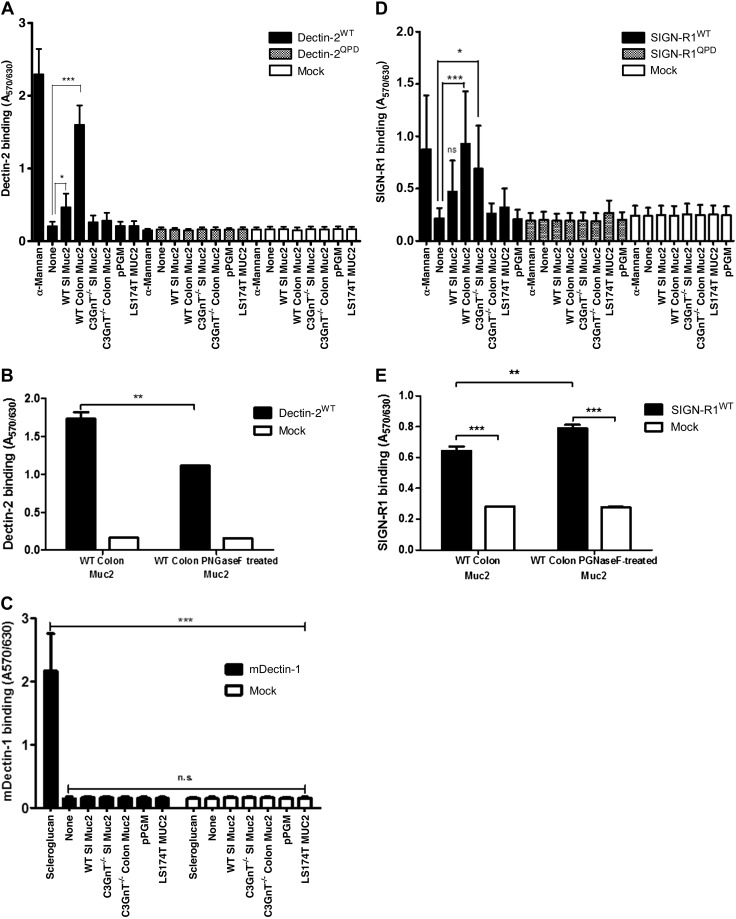
Interaction of mucins with CLRs. Cell reporter assays were performed to screen the interaction of SI or colon Muc2 from WT and *C3GnT^−/−^* mice, pPGM (MUC6) and LS174T MUC2 with mDectin-2 and QPD-mutant reporter cells (*A*), mDectin-1 reporter cells (*C*), and SIGN-R1 and QPD-mutant reporter cells (*D*). *B*, *E*) Binding between native and PNGase F–treated WT colon Muc2 with mDectin-2 reporter cells and SIGN-R1 reporter cells, respectively. *Saccharomyces cerevisiae* α-mannan was used as a positive control for mDectin-2 and SIGN-R1, and scleroglucan for mDectin-1, respectively. Mucins were used at 100 μg/ml, scleroglucan at 1 μg/ml, α-mannan at 2 μg/ml, and 100 μl of each was used for immobilization in the wells. Data are representative of 3 independent experiments with similar results. Error bars denote the sd. *P < 0.05, **P < 0.01, ***P < 0.001 (by ANOVA).

**Figure 7.**
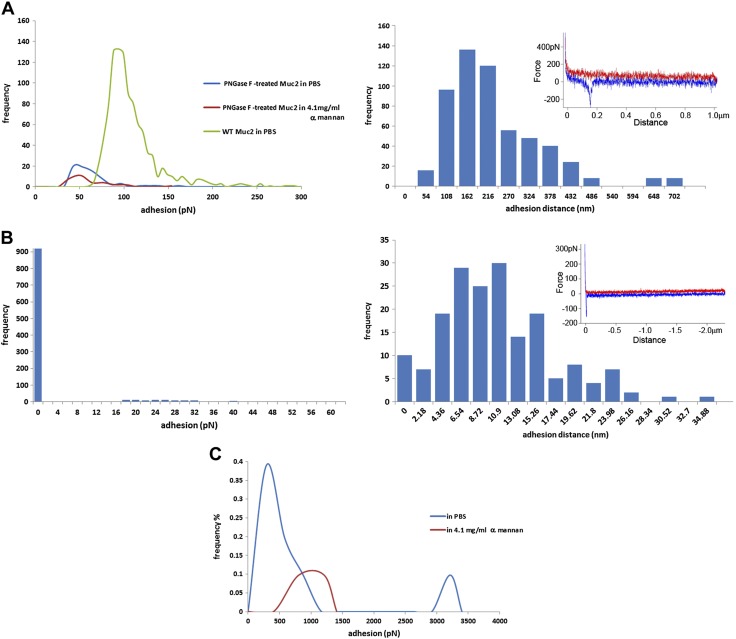
Force spectra histograms of adhesion between murine C-type lectins and mucin. *A*) Left: mucin adhesion quantification of mDectin-2 against WT colonic Muc2 or PNGase F–treated Muc2; right: mDectin-2- WT colonic Muc2 tip–sample adhesion separation distances. *B*) Left: mDectin1–pPGM adhesion quantification; right: mDectin1–pPGM tip–sample adhesion separation distances. Insets in right panels: examples of force–distance curves. *C*) SIGN-R1-WT colonic Muc2. Colonic Muc2 was covalently attached to the glass slide, C-type lectins were attached to the AFM tip *via* the PEG linker. Competition with *S. cerevisiae* α-mannan is included in the adhesion histograms.

**Figure 8.**
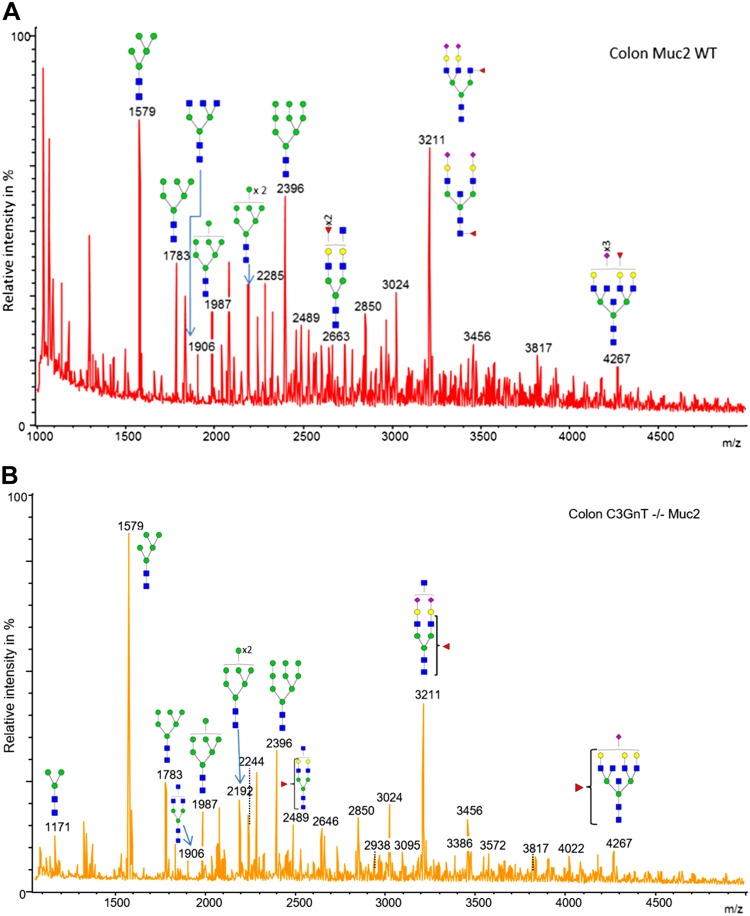
Structural characterization of mouse colonic Muc2 *N*-glycans. MALDI-MS spectra, acquired in the positive ion mode [M+Na]^+^ of permethylated *N*-glycans from colonic Muc2 from WT (*A*) and *C3GnT^−/−^* (*B*) mice after treatment by trypsin and PNGase F. Monosaccharide symbols follow the Symbol Nomenclature for Glycans system (81). Key: fucose (red triangle), GlcNAc (blue square), sialic acid (purple diamond), *N*-glycolyl sialic acid (light blue diamond), galactose (yellow circle), and mannose (green circle).

Using reporter cells expressing individual CLRs on the cell surface (42, 43), we assessed the binding of a range of mucins to mDectin1, mDectin-2, and SIGN-R1 (**Fig. 6**). We showed a significant binding of WT SI and colonic Muc2 to mDectin-2, whereas less binding was detected for *C3GnT^−/−^* Muc2 (SI and colon), pPGM, and LS174T MUC2 (Fig. 6*A*). There was no binding of mucins to mock cells (used a negative control) and the binding of WT SI and colonic Muc2 was reduced when mucin was incubated with reporter cells expressing the mDectin-2^QPD^ mutant in which the mannose-binding activity was eliminated by substituting glutamic acid-proline-asparagine sequence into the galactose-type QPD, indicating the specificity of the interaction. Dectin-2 CRD recognizes high-mannose ligands found in diverse microbes (67). AFM confirmed direct interaction between mDectin-2 and WT colonic Muc2, showing a total number of adhesion events of 1018 (**Fig. 7*A***). In addition, the large range of adhesion distance events revealed that the interactions occur along the entire length of the Muc2 chain as illustrated by the example force curve. To determine whether binding of mDectin-2 to mucins is mediated by *N*-linked high mannose of mucins, we carried out *N*-glycan profiling of colonic Muc2 from WT by MS. Reduced and alkylated mucins were first digested by trypsin before PNGase F treatment to allow release of *N*-glycans. We showed that WT colonic Muc2 from mice harbored high-mannose *N*-glycans from Man5GlcNAc2 at *m*/*z* = 1579 to Man9GlcNAc2 at *m*/*z* = 2396 (**Fig. 8*A*** and Supplemental Table S2). Note that a single *N*-glycan of the high-mannose type was reported in the recombinant human MUC2 C-terminus produced in CHO-K1 cells (68). A marked decrease in binding was detected with PNGase F–treated colonic Muc2, as shown in an mDectin-2 reporter cell assay (Fig. 6*B*) and force spectroscopy (Fig. 7*A*). The total number of adhesion events dropped from 1018 with Muc2 to 72 when mDectin-2 was assayed against PNGase F–treated Muc2. Both the modal values of adhesion event frequencies and of adhesion magnitude decreased against PNGase F–treated Muc2 (20 events of 43 pN), as compared to nontreated Muc2 (133 events of 94 pN). PNGase F treatment alone did not fully abolish the binding that may be related to a partial removal of *N*-glycans, the possible contribution of additional factors including other glycan epitopes, or both. However, the addition of yeast α-mannan to the liquid cell caused a further reduction of the modal adhesion event frequencies (11) and the total number of adhesion events dropped to 32, further supporting that the interaction occurs between mDectin-2 CRD and *N*-glycosylation epitopes in Muc-2. Oligomannosylated *N*-glycans were also present in *C3GnT^−/−^* Muc2 (Fig. 8*B* and Supplemental Table S2). However, we cannot exclude that fewer glycan structures may be responsible for the observed differences in binding between WT and *C3GnT^−/−^* colon Muc2. On the other hand, although the family of oligomannosylated *N*-glycans from Man5GlcNAc2 to Man9GlcNAc2 were clearly detected in spectra of colon Muc2 *N*-glycans, only oligomannosylated *N*-glycans at *m*/*z* = 1783 and 1987 were found in pPGM with fucosylated *N*-glycans representing the major structures (data not shown), which could explain the low interaction observed between pPGM and mDectin-2.

In contrast to the significant binding of WT SI and colonic Muc2 to mDectin-2, we did not detect any binding of mucin to mDectin-1 *via* the cell reporter assay (Fig. 6*C*). This lack of binding between mDectin-1 and mucin was further confirmed by force spectroscopy (Fig. 7*B*). The PEG linker used in the tip functionalization allows discrimination of specific and nonspecific adhesion events in force curves (69). Occasional nonspecific adhesions because of attractions between the tip and the sample surface occurs at the tip–sample detachment point, which is 0 nm on the *x*-axis and the gradient is linear, matching the bending of the AFM cantilever. We confirmed that the small number of adhesion events in the force spectra data of mDectin-1 against mucin (Fig. 7*B*) were nonspecific, as is clearly shown by the example force curve in the inset. This result is in marked contrast to the force measurements between mDectin-2 and mucin (Fig. 7*A*). These data are consistent with those in studies showing specificity of Dectin-1 for β-1,3-glucan, a polymer of glucose present in the fungal cell wall (70–72) and unlikely to recognize mucin glycans.

Colonic Muc2 from WT mice and intestinal Muc2 from *C3GnT^−/−^* mice interacted with SIGN-R1 in reporter cell assays (Fig. 6*D*). No binding was detected to mock cells (negative control) or SIGN-R1-QPD, supporting the specificity of the binding to the CRD of SIGN-R1. Although SIGN-R1 has also been shown to bind to mannose structures in fungal and other pathogens (65), treating colonic Muc2 with PNGase F did not reduce the binding to SIGN-R1 reporter cells (Fig. 6*E*). SIGN-R1 can also bind to fucose (73), and it is possible that binding to Muc2 is *via* fucosylated *O*-glycans (Fig. 3*D*). The high-resolution crystal structures of SIGN-R1 CRD in complex with sialylated ligands revealed the presence of an additional binding site, which may allow SIGN-R1 to simultaneously bind both immune glycoproteins and microbial polysaccharide components (74). Figure 7*C* shows the histogram of the interactions between SIGN-R1 and colonic Muc2, as measured by force spectroscopy. The modal value of adhesions occurred mostly at 292 pN but some binding events also occurred at 3212 pN. Addition of yeast α-mannan caused some reduction in interactions adhesion events, but the modal value of adhesion remained high, at 1008 pN. However, there was only a low number of specific interaction events (8 in PBS and 2 after α-mannan addition). The frequency, presented as the percentage of the 1024 force curves performed in each experiment, illustrates the rarity of interactions. It is worth noting that binding was also observed between colonic WT Muc2 and human recombinant lectins, hDectin-2 and DC-SIGN, with similar adhesion magnitude range between these lectins, whereas interaction with hDectin-1 was reduced, in broad agreement with the results obtained with mouse lectins (Supplemental Fig. S5).

### CLRs, mDectin-1, mDectin-2, and SIGN-R1, are *N*-glycosylated and bind to Gal-3

Dectin-1 is glycosylated, a modification that contributes to its surface expression and function (75). One of the major differences among murine and human isoforms of Dectin-1 is the position and number of *N*-linked glycosylation motifs. mDectin-1 was previously reported to be *N*-glycosylated with terminal galactose sometimes capped with *N*-acetylneuraminic acid (76), but no structural information is available on the glycosylation of mDectin-2 or SIGN-R1. The deduced amino acid sequences of mDectin-2 from murine DC line XS52 indicate the presence of a putative *N*-glycosylation site in the CRD domain (77) whereas SIGN-R1 presents 2 *N*-glycosylation sites, according to Uniprot (*http://www.uniprot.org/uniprot/Q8CJ91*).

**Figure 9.**
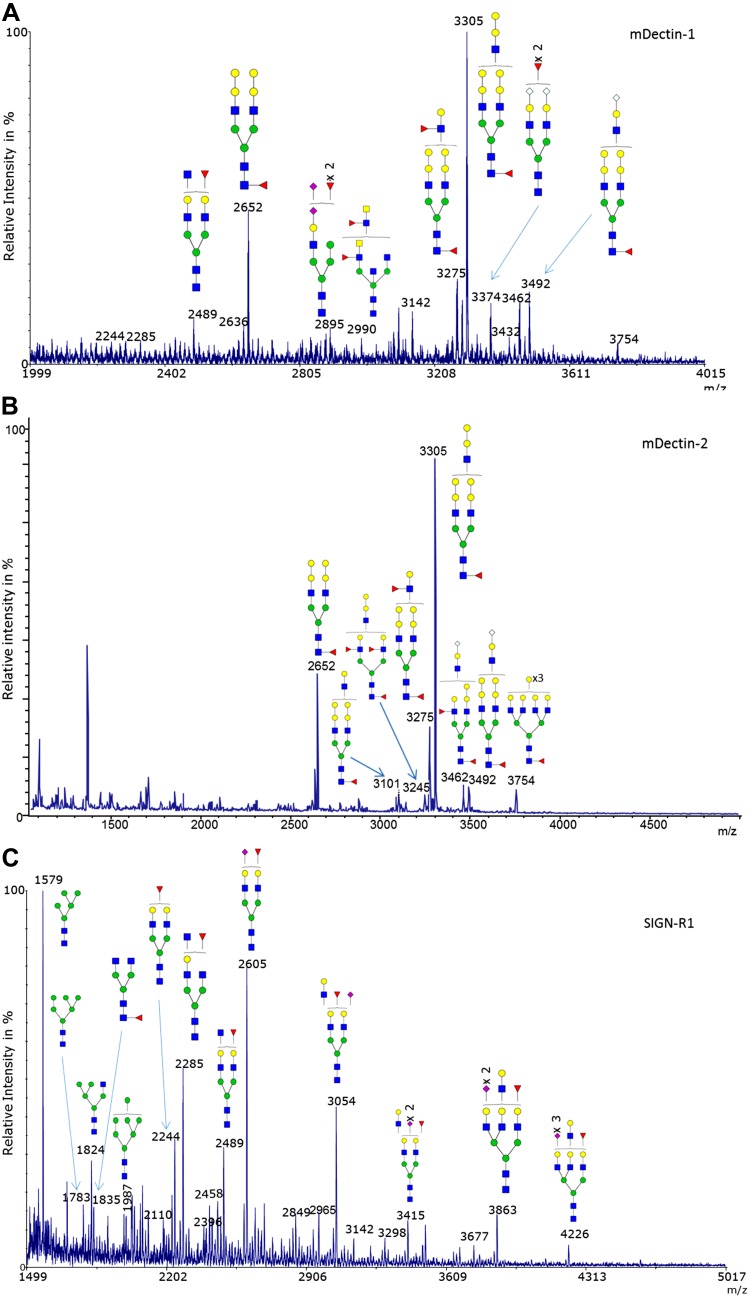
Structural characterization of mice C-type lectin *N*-glycans. MALDI-MS spectra, acquired in the positive ion mode [M+Na]^+^ of permethylated *N*-glycans from recombinant C-type lectins from mDectin-1 (*A*), mDectin-2 (*B*), and SIGN-R1 (*C*). Monosaccharide symbols follow the Symbol Nomenclature for Glycans system (81). Key: fucose (red triangle), GlcNAc (blue square), sialic acid (purple diamond), *N*-glycolyl sialic acid (light blue diamond), galactose (yellow circle), and mannose (green circle).

**Figure 10.**
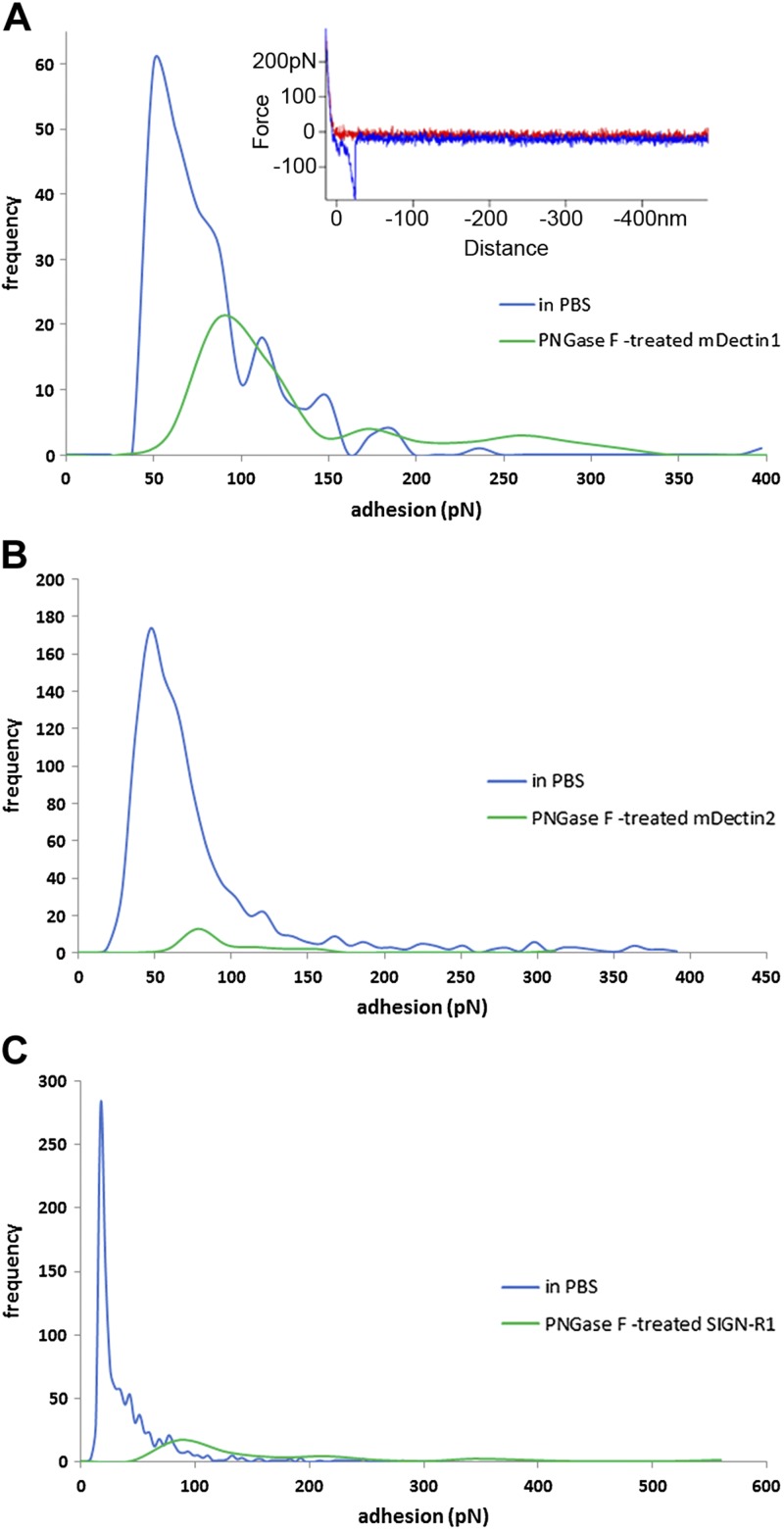
Force spectra histograms of adhesion between Gal-3 and murine C-type lectins. *A*) Interaction between Gal-3 and untreated or PNGase F–treated mDectin-1. Gal-3 was attached to the AFM tip and mDectin-1 to a slide. Inset: example force–distance curve. *B*) Interaction between Gal-3 and untreated or PNGase F–treated mDectin-2. (*C*) Interaction between Gal-3 and untreated or PNGase F–treated SIGN-R1. Gal-3 was attached to a slide and mDectin-2 or SIGN-R1 on the AFM tips.

To gain further structural insights into the nature of glycans decorating C-type-lectins, we performed MS analysis of recombinant mDectin-1, mDectin-2, and SIGN-R1. All 3 C-type lectins were found to be *N*-glycosylated. mDectin-1 and mDectin-2, both produced in the NSO cell line derived from nonsecreting murine myeloma, showed similar *N*-glycan structures (Supplemental Table S3). Two major *N*-glycans at *m*/*z* = 2652 and *m*/*z* = 3305 were observed in mDectin-1 and mDectin-2, corresponding to fucosylated structures with 2 and 3 antennae terminated by 2 Gal residues (**Fig. 9*A, B*** and Supplemental Fig. S6). SIGN-R1 presented oligomannosylated and complex-type bi-,tri-antennary *N*-glycans, with major glycans at *m*/*z* = 1579 (oligomannosylated glycans), *m*/*z* = 2244, *m*/*z* = 2285, *m*/*z* = 2489, *m*/*z* = 2605, and *m*/*z* = 3054 which are fucosylated, capped with Neu5Ac, or both. (Fig. 9*C*). These *N*-glycans found in mDectin-1, mDectin-2, and SIGN-R1 could represent potential ligands for Gal-3, in particular the potential β-1,6-*N*-acetylglucosamine antenna present in all 3 C-type lectins. To test this hypothesis, these CLRs were tested for their binding to Gal-3 by force spectroscopy. Previous work showed that Gal-3 was involved in an interaction with Dectin-1-FcγRIIB-MUC2 complex on DCs (32). However, the detailed binding properties have not been clarified and structurally characterized. We showed that Gal-3 recognizes mDectin-1, mDectin-2, and SIGN-R1 *via* a carbohydrate–lectin interaction. Force spectroscopy measurements showed specific interactions between Gal-3 and these ligands (**Fig. 10**). The inset example force–distance curves in Fig. 10*A* illustrates that the adhesion events between the 2 discrete globular proteins, Gal-3, and mDectin-1, is singular (*i.e.*, not multiple), in contrast to the multiple and long-range adhesion events seen when Gal-3 interacts with mucin. The modal value of the adhesive interactions between Gal-3 and mDectin-1 was 49.68 pN. Figure 10*B*, *C* shows the interaction between Gal-3 attached to a slide and mDectin-2 or SIGN-R1 on AFM tips, respectively. The modal value of adhesion between Gal-3 and mDectin-2 was 46.55 pN, whereas the modal value of adhesion between Gal-3 and SIGN-R1 was 17.08 pN, PNGase F-treatment significantly reduced the frequency of interactions, further supporting that binding of Gal-3 to these C-type lectins is *via* *N*-linked β-galactose structures decorating recombinant mDectin-1, mDectin-2, and SIGN-R1. These results are also in line with the reported high affinity of Gal-3 for β-1,6-N-GlcNAc branched glycans (42).The importance of glycosylation on the biologic function of some CLRs has been reported, including E-selectin (78), LOX-1 (79), class I major histocompatibility complex–binding receptors (80), and more recently Dectin-1 (75). The comprehensive structural characterization of the *N*-glycans decorating mDectin-1, mDectin-2, and SIGN-R1, as reported here, provides a basis for mechanistic follow-up studies. The glycosylation of C-type lectins in myeloid and nonmyeloid cells may play a key and unrecognized role in mediating crucial cellular functions during immunity and homeostasis, and a potential mechanism for the clustering of C-type lectins and Gal-3 *via* the recognition of glycans on self-proteins.

## Supplementary Material

This article includes supplemental data. Please visit *http://www.fasebj.org* to obtain this information.

## Abbreviations

AFM: atomic force microscopy
C3GnT: core-3 β1,3-*N*-acetylglucosaminyltransferase
CLR: C-type lectin receptor
CRD: carbohydrate recognition domain
DC: dendritic cell
DC-SIGN: dendritic cell-specific intercellular adhesion molecule-3–grabbing nonintegrin
dectin: dendritic cell-associated lectin
Gal-3: galectin-3
GalNAc: *N*-acetylgalactosamine
GC: goblet cell
GlcNAc: *N*-acetylglucosamine
GI: gastrointestinal
LacNAc: *N*-acetyllactosamine
MAL: maleimide
MALDI-TOF: matrix-assisted laser desorption and ionization–time of flight
MS/MS: tandem mass spectrometry
PEG: polyethylene glycol
PNGase F: peptide:*N*-glycosidase F
PGM: porcine gastric mucin
pPGM: purified porcine gastric mucin
RCA: *Ricinus communis* agglutinin
SCM: succinimidyl carboxyl methyl ester
SI: small intestine
SIGN-R: specific intercellular adhesion molecule–grabbing nonintegrin-related
TF: Thomsen-Friedenreich
WGA: wheat germ agglutinin, UEA, *Ulex europaeus* agglutinin
WT: wild type
