# Supplementary material for: Molecular basis for intestinal mucin recognition by galectin-3 and C-type lectins
Source: FASEB J. 2018 Jan 29;32(6):3301–20. doi: 10.1096/fj.201700619R (PMC5976236; doi:10.1096/fj.201700619R)
Supplement: Supplementary file 1 [file fj.201700619R.sd1.pdf]

Supplemental data

Figure S1 Separation of the guanidinium chloride insoluble mucins by SDS-PAGE (small proteins).

Protein were detected by (**A**) Coomassie blue, (**B**) anti-MUC2, (**C**) WGA, (**D**) Alcian blue staining. The Coomassie blue staining shows the total proteins in the samples. 1- HiMark protein standard (Invitrogen, Thermo Fisher Scientific), 2-Fetuin (glycoprotein control), 3- BSA (glycosylation negative control), 4-WT SI Muc2, 5-*C3GnT*$^{-/-}$ SI Muc2, 6- WT colon Muc2, 7- *C3GnT*$^{-/-}$ colon Muc2, 8-pPGM, 9- LS174T MUC2

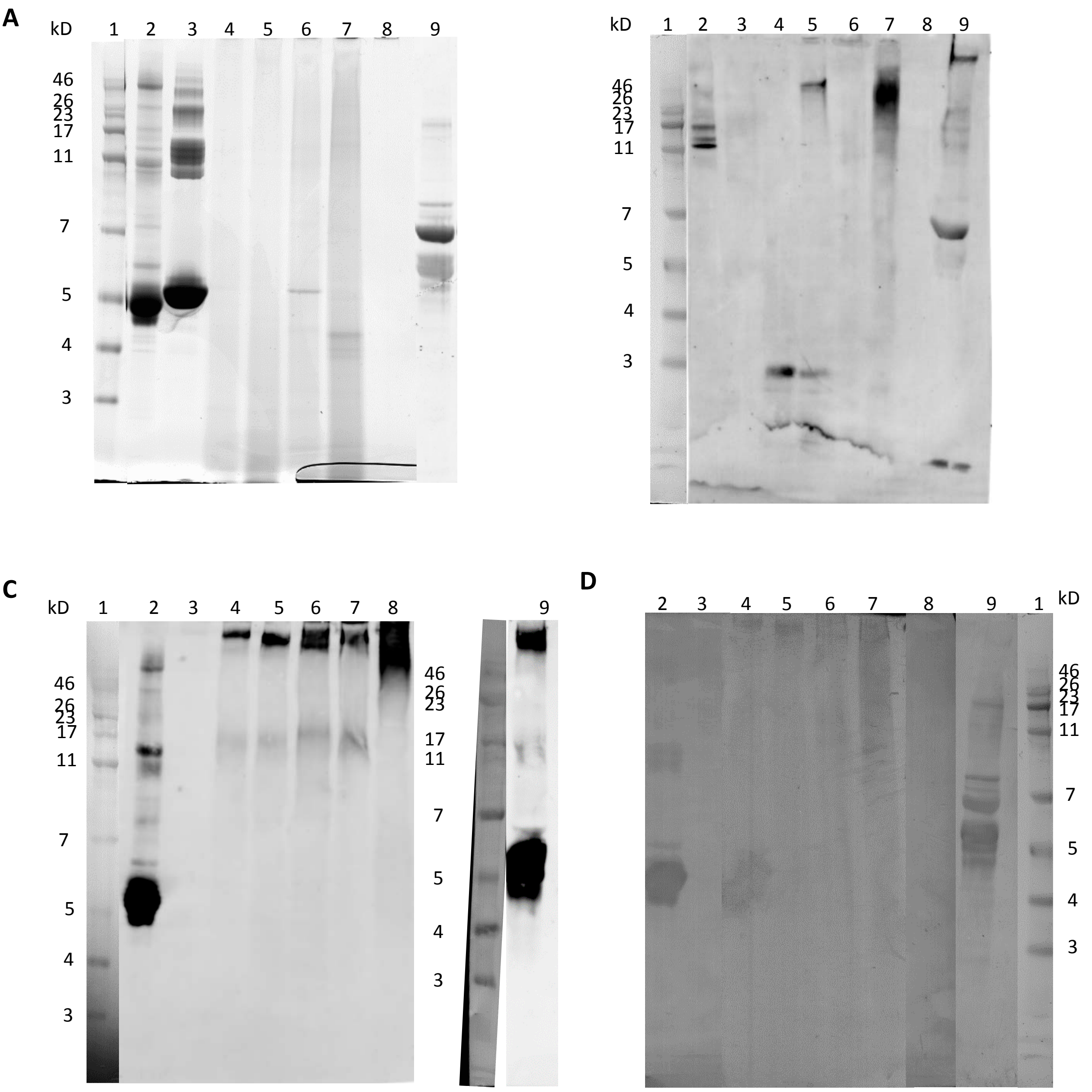

Figure S2 Separation of the guanidinium chloride insoluble mucins by VAGE (high molecular weight proteins). Proteins were detected by (**A**) anti-MUC2, (**B**) WGA, (**C**) Alcian blue staining. 1- HiMark protein standard, 2-Fetuin (glycoprotein control), 3- BSA (glycosylation negative control), 4-WT SI Muc2, 5-*C3GnT*$^{-/-}$ SI Muc2, 6- WT colon Muc2, 7- *C3GnT*$^{-/-}$ colon Muc2, 8-pPGM, 9- LS174T MUC2

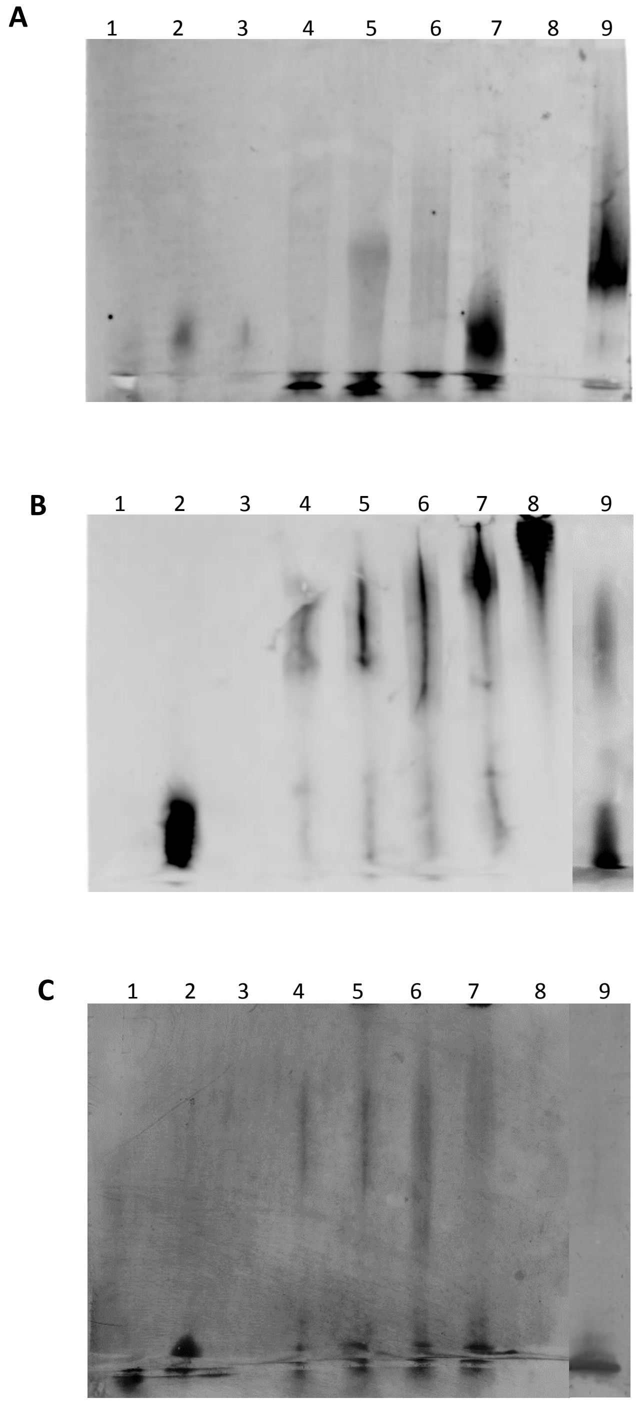

Figure S3 MS/MS spectra of *O*-glycans from mice Muc2.

O-glycans were fragmented in positive mode from (**A**) colon WT and *C3GnT*$^{-/-}$ mice and (**B**) from small intestine WT and *C3GnT*$^{-/-}$ mice. Monosaccharide symbols follow the Symbol Nomenclature for Glycans (SNFG) system (1). Key: fucose (red triangle), GlcNAc (blue square), sialic acid (purple diamond), galactose (yellow circle) and HexNAc (off-white square).

A

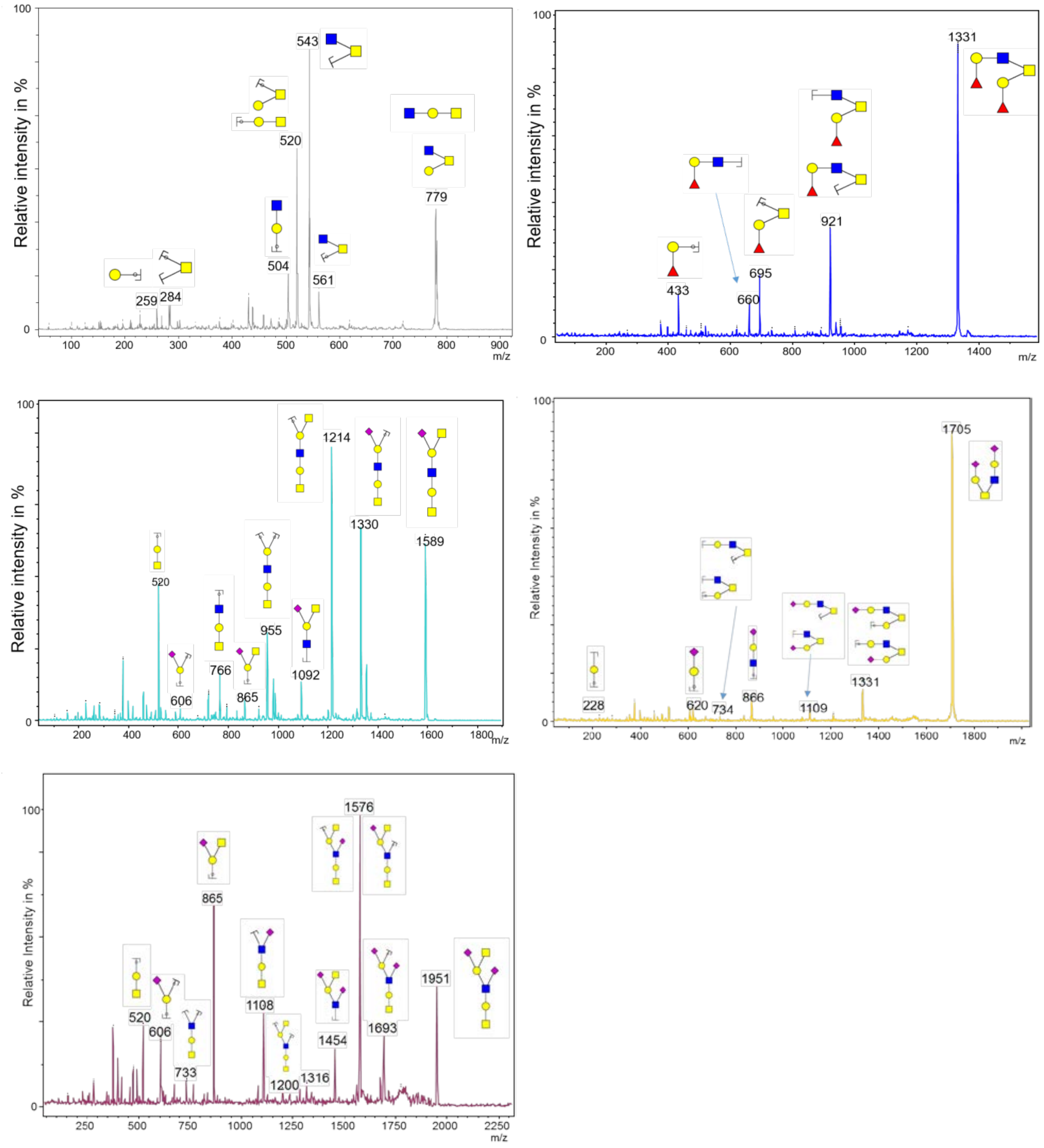

B

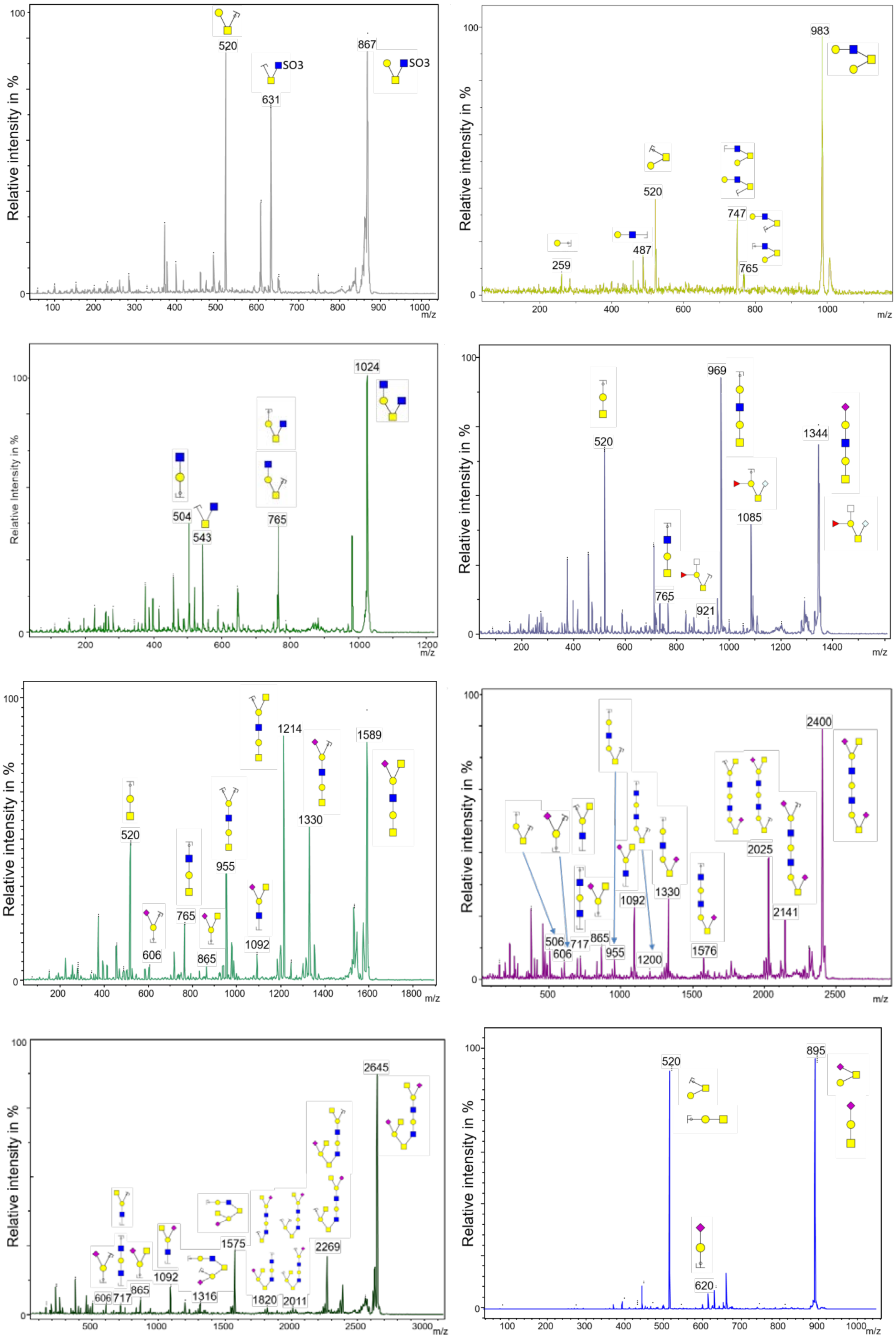

Relative intensity in %
100
0
520
631
867
SO3
SO3
m/z
983
259
487
520
747
765
1024
504
543
765
969
520
765
921
1085
1344
1214
1589
520
606
765
865
955
1092
1330
2400
506
606
717
865
955
1092
1200
1330
1576
2025
2141
2645
606
717
865
1092
1316
1575
1820
2011
2269
520
620
895

Figure S4 Structural characterisation of pPGM *O*-glycan structures after deglycosylation cycles

MALDI-MS spectra, acquired in the positive ion mode [M + Na]$^+$ of permethylated *O*-glycans from pPGM after **(A)** one cycle of deglycosylation and **(B)** two cycles of deglycosylation. (**C**) shows a histogram of the different *O*-glycans found on pPGM after several cycles of deglycosylation. The proportion of each *O*-glycan is relative to other species in a sample Monosaccharide symbols follow the Symbol Nomenclature for Glycans (SNFG) system (1). Key: fucose (red triangle), GlcNAc (blue square), sialic acid (purple diamond), N-glycolyl sialic acid (light blue diamond), galactose (yellow circle), and HexNAc (off-white square).

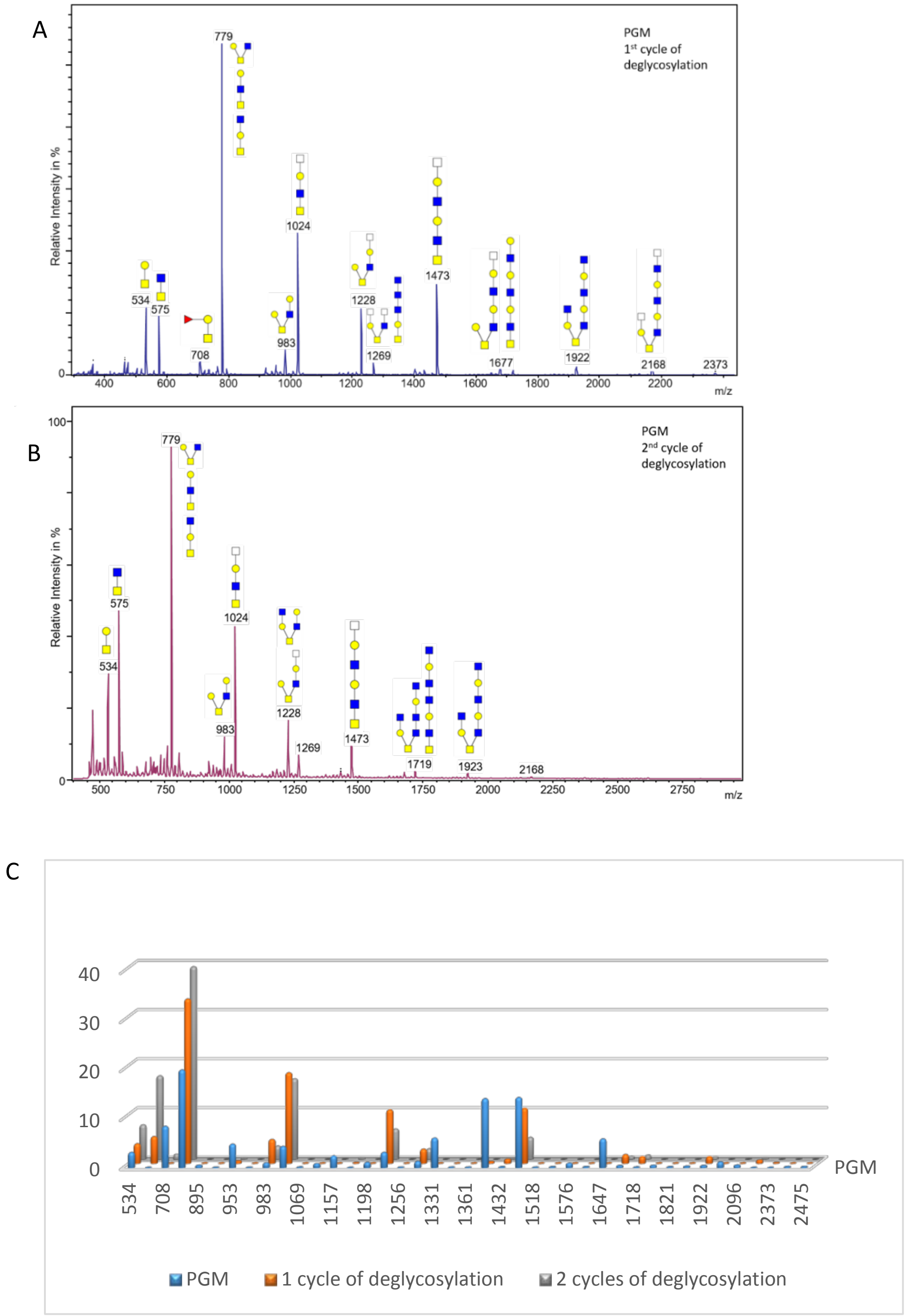

Figure S5 Force spectra histograms of adhesion between human C-type lectins and mucin. Interaction between WT colonic Muc2 and (**A**) hDectin-1, (**B**) hDectin-2, (**C**) DC-SIGN in presence or absence of potential inhibitors.

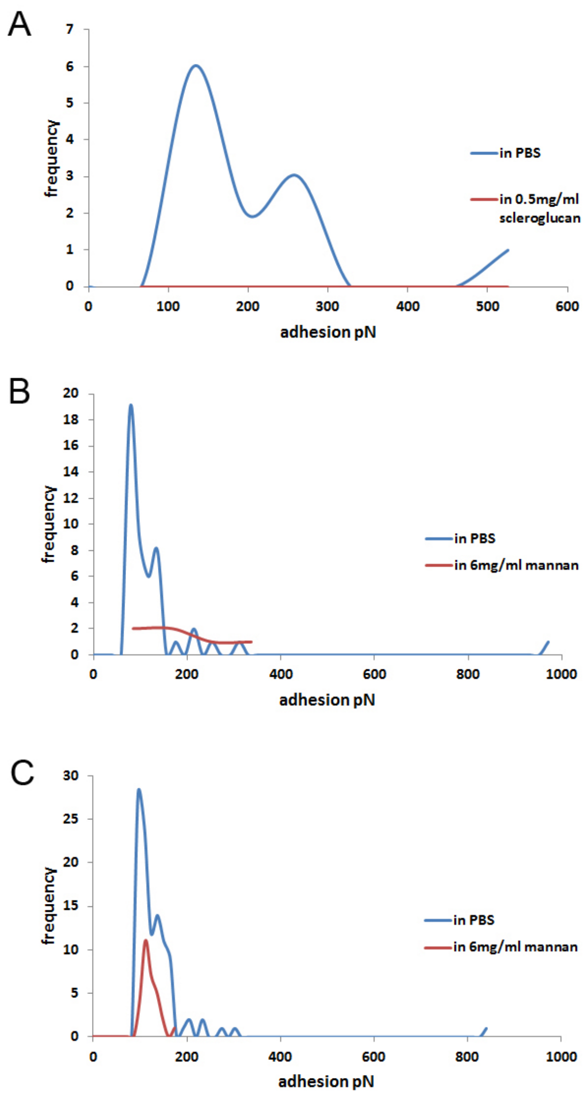

Figure S6: MS/MS spectra of *N*-glycans from mDectin-2.

MS/MS spectra of parent ion at m/z=2652; 3275 and 3305 fragmented in positive mode. Monosaccharide symbols follow the Symbol Nomenclature for Glycans (SNFG) system (1). Key: fucose (red triangle), GlcNAc (blue square), sialic acid (purple diamond), galactose (yellow circle) and HexNAc (off-white square).

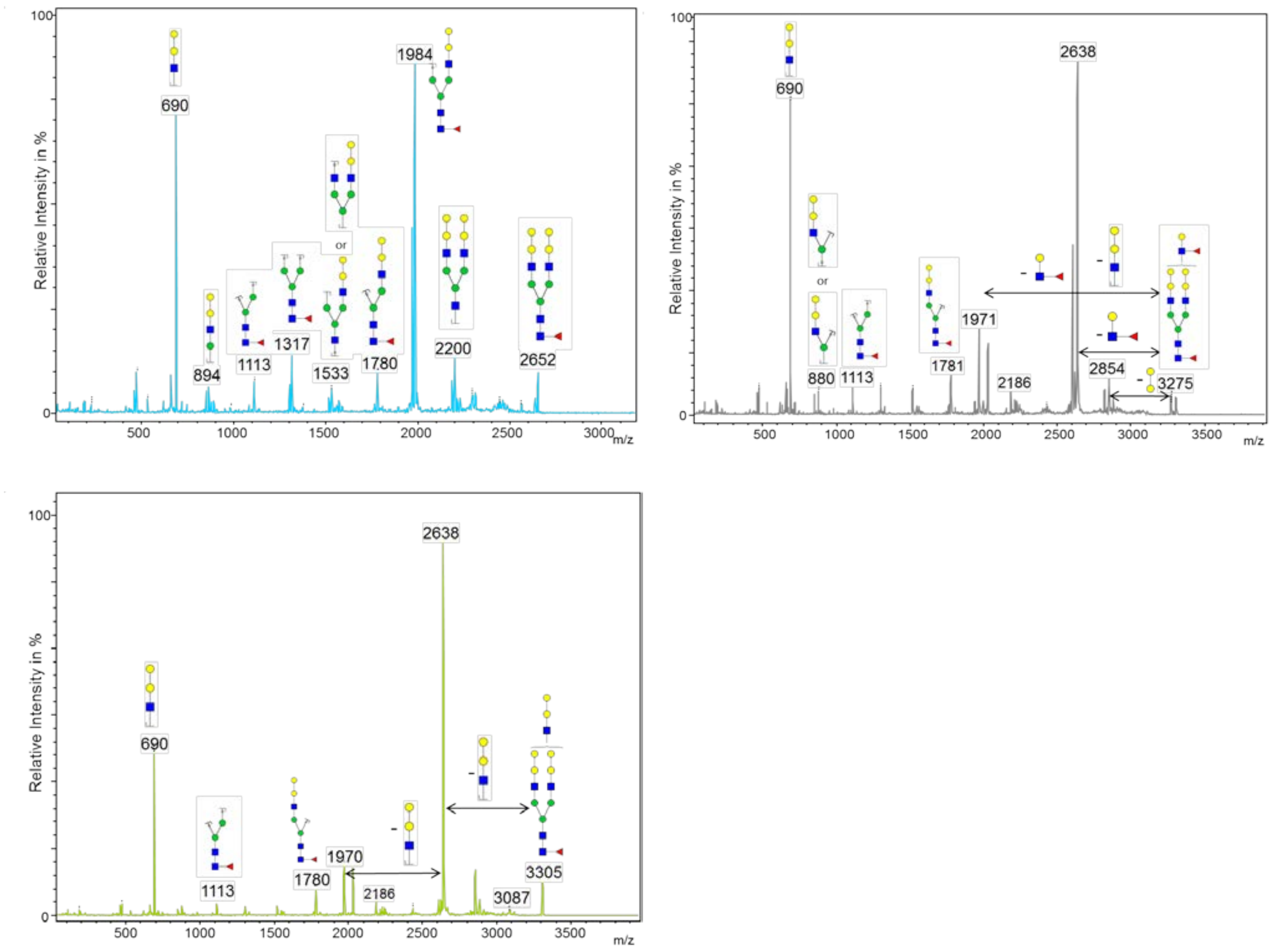

Table S1 Proposed pPGM *O*-glycan structures after deglycosylation cycles. Quantification through the relative percentage of each oligosaccharide was calculated based on integration of peaks on MS spectra.

		pPGM	pPGM 1st cycle	pPGM 2nd cycle
Sequence of oligosaccharides	**mass**	**relative %**	**relative %**	**relative %**
1 Gal, GalNAcol	534	3.18 +/-1.29	4.06 +/-3.25	7.03 +/-1.93
HexNAc, GalNAcol	575		5.53 +/-5.66	17.02 +/-8.48
1 Gal, 1 Fuc, GalNAcol	708	8.51 +/-2.12		1.04 +/- 0.74
1 Gal, 1 HexNAc, GalNAcol	779	19.99 +/-6.69	33.61 +/-2.67	39.30 +/-5.74
1 Gal, 1 NeuAc, GalNAcol	895	0.55 +/-0.19		
2 Gal, 1 Fuc, GalNAcol	912	0.08 +/-0.12		
1 Gal, 1 HexNAc, 1 Fuc, GalNAcol	953	4.87 +/-0.81	0.57 +/-0.24	0.09 +/-0.12
NeuGc,HexNAc,GalNAcol	967	0.09 +/-0.13		
2 Gal, 1 HexNAc, GalNAcol	983	1.06 +/-0.33	4.95 +/-1.52	2.70 +/-0.58
1 Gal, 2 HexNAc, GalNAcol	1024	4.42 +/-0.34	18.55 +/-1.86	16.43 +/-5.44
1 Gal, 1 Fuc, 1 NeuAc, GalNAcol	1069	0.14 +/-0.20		
1 Gal,1 HexNAc, 1 NeuAc, GalNAcol	1140	0.91 +/- 0.34		
2 Gal, 1 HexNAc, 1 Fuc, GalNAcol	1157	2.49 +/-0.84		
3 Gal, 1 HexNAc, GalNAcol	1187		0.15 +/-0.22	0.20 +/-0.28
1 Gal, 2 HexNAc, 1 Fuc, GalNAcol	1198	1.14 +/-0.31		
2 Gal, 2 HexNAc, GalNAcol	1228	3.19 +/-0.75	11.00 +/-1.22	6.18 +/-3.54
1 Gal, 2 NeuAc, GalNAcol	1256	0.10 +/-0.14		
1 Gal, 3 HexNAc, GalNAcol	1269	1.36 +/-0.22	2.98 +/-1.08	
2 Gal, 1 HexNAc, 2 Fuc, GalNAcol	1331	6.11 +/-2.18		2.13 +/-1.30
2 Gal, 1 HexNAc, 1 NeuAc, GalNAcol	1344	0.12 +/-0.14		
3 Gal, 1 HexNAc, 1 Fuc, GalNAcol	1361	0.11 +/-0.15		
2 Gal, 2 HexNAc, 1 Fuc, GalNAcol	1402	14.14 +/-0.73	0.44 +/-0.62	
3 Gal, 2 HexNAc, GalNAcol	1432	0.19 +/-0.27	0.96 +/-0.88	0.17 +/-0.24
2 Gal, 3 HexNAc, GalNAcol	1473	14.38 +/-2.84	11.30 +/-6.20	4.47 +/-3.06
2 Gal, 1 HexNAc, 1 Fuc, 1 NeuAc, GalNAcol	1518	0.11 +/-0.15		
3 Gal, 1 HexNAc, 2 Fuc, GalNAcol	1535	0.21 +/-0.30		
2 Gal, 2 HexNAc, 2 Fuc, GalNAcol	1576	1.00 +/-0.72		
3 Gal, 2 HexNAc, 1 Fuc, GalNAcol	1606	0.24 +/-0.34		
2 Gal, 3 HexNAc, 1 Fuc, GalNAcol	1647	5.94 +/-0.96		
3 Gal, 3 HexNAc, GalNAcol	1677	0.49 +/-0.38	1.89 +/-0.72	0.65 +/-0.49
2 Gal, 4 HexNAc, GalNAcol	1718	0.14 +/-0.20	1.46 +/-0.73	0.83 +/-0.63
3 Gal, 2 HexNAc, 2 Fuc, GalNAcol	1780	0.54 +/-0.43		
2 Gal, 3 HexNAc, 2 Fuc, GalNAcol	1821	0.22 +/-0.31		
3 Gal, 3 HexNAc, 1 Fuc, GalNAcol	1851	0.35 +/-0.49		
3 Gal, 4 HexNAc, GalNAcol	1922	0.51 +/-0.37	1.45 +/-0.05	0.52 +/-0.38
3 Gal, 3 HexNAc, 2 Fuc, GalNAcol	2025	1.26 +/-0.28		
3 Gal, 4 HexNAc, 1 Fuc, GalNAcol	2096	0.58 +/-0.31		
3 Gal, 5 HexNAc, GalNAcol	2168		0.75 +/-0.57	0.18 +/-0.25
4 Gal, 3 HexNAc, 3 Fuc, GalNAcol	2404	0.27 +/-0.27		
4 Gal, 5 HexNAc, GalNAcol	2373		0.15 +/-0.22	
4 Gal, 4 HexNAc, 2 Fuc, GalNAcol	2475	0.26 +/-0.19		

Table S2 Proposed oligosaccharide structures of colonic WT and *C3GnT*$^{-/-}$ Muc2 *N*-glycans. Reduced and alkylated mucins were digested by trypsin and then treated by PNGase F to release *N*-glycans. Quantification through the relative percentage of each oligosaccharide was calculated based on the integration of peaks from the MS spectra.

		Relative %	
Proposed structures or sequence of oligosaccharides	***[M+Na]$^+$***	***MUC2 Colon C3GnT-/-***	***MUC2 Colon WT***
**3 Man, 2 GlcNAc** 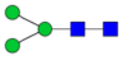	1171	0,92	-
**3 Man, 1 Fuc, 2 GlcNAc** 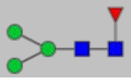	1345	1,10	-
**5 Man, 2 GlcNAc** 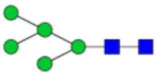	1579	10,12	5,57
**3 Man, 1 Fuc, 3 GlcNAc** 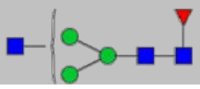	1590	-	1,11
**6 Man, 2 GlcNAc** 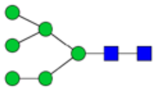	1783	6,38	3,83
**3 Man, 1 Fuc, 4 GlcNAc** 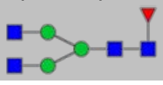	1835	2,25	3,20
**3 Man, 5 GlcNAc** 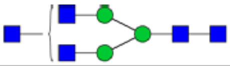	1906	-	1,53
**7 Man, 2 GlcNAc** 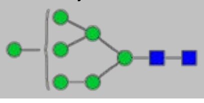	1987	3,87	3,05
**1 Gal, 3 Man, 1 Fuc, 4 GlcNAc** 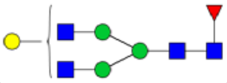	2040	2,09	2,12
**2 Gal, 3 Man, 4 GlcNAc** 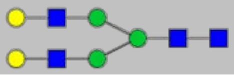	2070	1,80	-
**1 Gal, 3 Man, 5 GlcNAc** 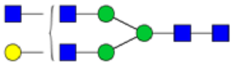	2111	1,49	1,61
**8 Man, 2 GlcNAc** 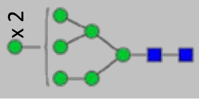	2192	5,59	4,17
**2 Gal, 3 Man, 1 Fuc, 4 GlcNAc** 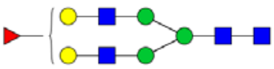	2244	4,01	3,10

1 Gal, 3 Man, 1 Fuc, 5 GlcNAc 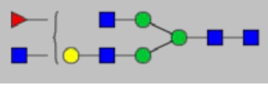	2285	5,13	4,28
9 Man, 2 GlcNAc 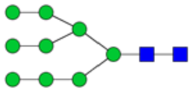	2396	6,91	7,16
2 Gal, 3 Man, 1 NeuAc, 4 GlcNAc 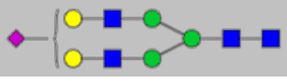	2431	1,30	-
1 Gal, 5 Man, 1 Fuc, 4 GlcNAc (*hybrid*)	2448	1,46	-
1 Gal, 3 Man, 2 Fuc, 5 GlcNAc 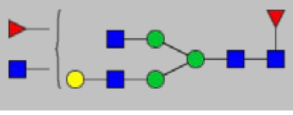	2459	1,87	-
2 Gal, 3 Man, 1 Fuc, 5 GlcNAc 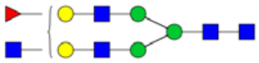	2489	3,06	3,13
1 Gal, 3 Man, 1 Fuc, 6 GlcNAc 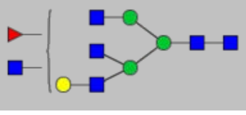	2530	-	2,69
3 Man, 1 Fuc, 5 HexNAc, 2 GlcNAc	2571	-	2,34
2 Gal, 3 Man, 1 NeuAc, 1 Fuc, 4 GlcNAc (*biantennary monosialylated and fucosylated*) 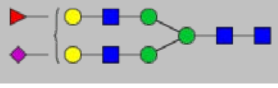	2605	2,45	2,07
5 Hex, 1 Fuc, 1 NeuGc, 4 GlcNAc/ 6 Hex, 1 NeuAc, 4 GlcNAc (*hybrid*) 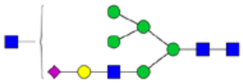	2635	2,53	-
1 Gal, 3 Man, 1 NeuAc, 1 Fuc, 5 HexNAc	2646	2,11	2,38
2 Gal, 3 Man, 2 Fuc, 5 GlcNAc 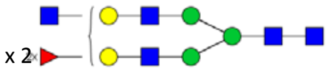	2663	1,69	2,25
2 Gal, 3 Man, 1 Fuc, 6 GlcNAc	2734	1,62	3,13
1 Gal, 3 Man, 1 Fuc, 5 HexNAc, 2 GlcNAc	2775	-	2,07
2 Gal, 3 Man, 3 Fuc, 6 GlcNAc 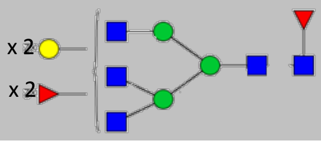	2837	2,00	-
2 Gal, 3 Man, 1 Fuc, 1 NeuAc, 5 GlcNAc	2850	-	3,88
2 Gal, 3 Man, 2 NeuAc, 1 Fuc, 4 GlcNAc (*biantennary disialylated and fucosylated*) 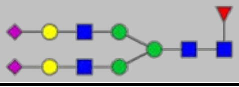	2966	2,62	3,49

**2 Gal, 3 Man, 1 Fuc, 5 HexNAc, 2 GlcNAc**	2979	-	2,34
**2 Gal, 3 Man, 2 Fuc, 1 NeuAc, 3 HexNAc, 2 GlcNAc**	3024	-	4,41
**3 Gal, 3 Man, 1 Fuc, 5 HexNAc, 2 GlcNAc**	3183	-	2,22
**2 Gal, 3 Man, 1 Fuc, 2 NeuAc, 5 GlcNAc** 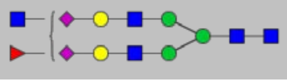	3211	8,27	10,31
**2 Gal, 3 Man, 1 Fuc, 6 HexNAc, 2 GlcNAc**	3224	-	2,79
**2 Gal, 3 Man, 1 Fuc, 2 NeuAc, 6 GlcNAc**	3456	5,23	3,57
**3 Gal, 3 Man, 1 Fuc, 1 NeuAc, 7 GlcNAc**	3544	2,95	-
**2 Gal, 3 Man, 1 Fuc, 3 NeuAc, 4 HexNAc, 2 GlcNAc**	3817	-	3,19
**1 Gal, 3 Man, 3 Fuc, 2 NeuGc, 7 GlcNAc/ 3 Gal, 3 Man, 1 Fuc, 2 NeuAc, 7 GlcNAc**	3905	3,52	-
**3 Gal, 3 Man, 1 Fuc, 3 NeuAc, 7 GlcNAc**	4267	5,70	2,99

Table S3 Proposed oligosaccharide structures of C-type lectins *N*-glycans. Quantification through the relative percentage of each oligosaccharide

			Relative %	
Proposed structures or sequence of oligosaccharides	***[M+Na]$^+$***	***mDectin-1***	***mDectin-2***	***SIGN-R1***
**5 Man, 2 GlcNAc** 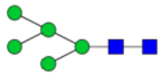	1579	-	-	14,94
**3 Man, 1 Fuc, 3 GlcNAc** 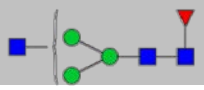	1590	-	-	1,70
**3 Man, 4 GlcNAc** 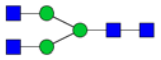	1661	-	-	1,37
**6 Man, 2 GlcNAc** 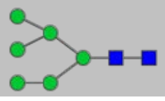	1783	-	-	2,23
**5 Man, 3 GlcNAc**	1824	-	-	4,09
**3 Man, 1 Fuc, 4 GlcNAc** 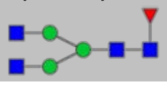	1835	-	-	2,30
**7 Man, 2 GlcNAc** 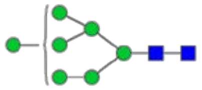	1987	-	-	1,56
**1 Gal, 3 Man, 1 Fuc, 4 GlcNAc** 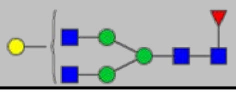	2040	1,46	-	2,78

1 Gal, 3 Man, 5 GlcNAc<br>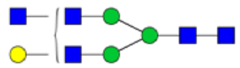	2110	-	-	2,83
8 Man, 2 GlcNAc<br>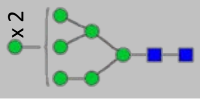	2192	-	-	1,41
2 Gal, 3 Man, 1 Fuc, 4 GlcNAc<br>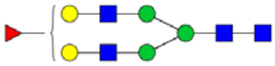	2244	1,42	-	5,05
1 Gal, 3 Man, 1 Fuc, 5 GlcNAc<br>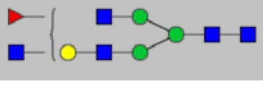	2285	-	-	7,54
9 Man, 2 GlcNAc<br>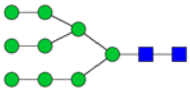	2396	-	-	0,86
1 Gal, 3 Man, 2 Fuc, 5 GlcNAc<br>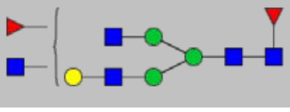	2459	-	-	2,56
2 Gal, 3 Man, 1 Fuc, 5 GlcNAc<br>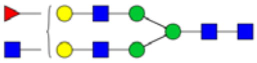	2489	5,16	-	4,89
3 Man, 2 Fuc, 2 HexNAc, 4 GlcNAc	2499	-	-	1,36
1 Gal, 3 Man, 1 Fuc, 6 GlcNAc<br>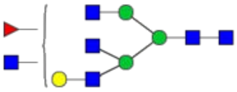	2530	-	-	1,14
3 Man, 1 Fuc, 5 HexNAc, 2 GlcNAc	2571	-	-	0,56
2 Gal, 3 Man, 1 NeuAc, 1 Fuc, 4 GlcNAc (*biantennary monosialylated and fucosylated*)<br>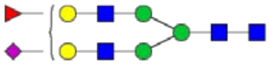	2605	0,93	-	11,28
1 Gal, 3 Man, 3 Fuc, 5 GlcNAc	2633	-	-	1,37
3 Gal, 3 Man, 1 NeuAc, 4 GlcNAc/ 2 Gal, 3 Man, 1 NeuGc, 1 Fuc, 4 GlcNAc	2636	5,96		
1 Gal, 3 Man, 1 NeuAc, 1 Fuc, 5 HexNAc	2646	-	-	1,80
4 Gal, 3 Man, 4 GlcNAc, 1 Fuc<br>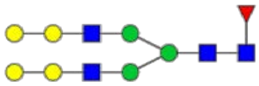	2652	15,48	17,50	-
2 Gal, 3 Man, 2 Fuc, 5 GlcNAc<br>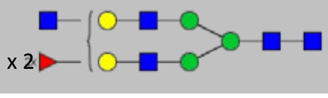	2663	-	-	1,54
2 Gal, 3 Man, 1 Fuc, 6 GlcNAc	2734	-	-	0,75
2 Gal, 3 Man, 2 Fuc, 1 NeuAc, 4 GlcNAc	2778	-	-	1,38

**2 Gal, 3 Man, 1 Fuc, 1 NeuAc, 5 GlcNAc**	2850	1,67	-	1,92
**4 Hex, 2 NeuAc, 2 Fuc, 3 GlcNAc**	2895	2,97	-	-
**2 Gal, 3 Man, 2 NeuAc, 1 Fuc, 4 GlcNAc (*biantennary disialylated and fucosylated*)** 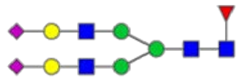	2966	-	-	1,95
**3 Man, 2 Fuc, 8 HexNAc**	2990	1,51	-	-
**3 Gal, 3 Man, 1 NeuAc, 1 Fuc, 5 GlcNAc**	3054	-	-	4,95
**5 Gal, 3 Man, 5 GlcNAc, 1 Fuc** 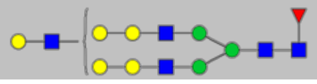	3101	5,93	2,64	-
**4 Gal, 3 Man, 1 Fuc, 6 GlcNAc**	3142	4,83	-	1,27
**4 Gal, 3 Man, 5 GlcNAc, 3 Fuc**	3245	-	1,83	-
**5 Gal, 3 Man, 5 GlcNAc, 2 Fuc** 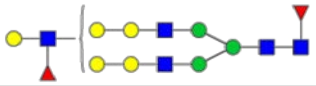	3275	10,26	11,93	-
**3 Gal, 3 Man, 1 Fuc, 1 NeuAc, 6 GlcNAc**	3298	-	-	0,80
**6 Gal, 3 Man, 5 GlcNAc, 1 Fuc** 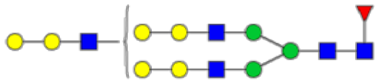	3305	22,90	54,22	-
**2 Gal, 3 Man, 3 Fuc, 2 NeuAc, 4 GlcNAc**	3314	-	-	0,62
**2 Gal, 3 Man, 2 NeuGc, 3 Fuc, 4 GlcNAc**	3374	4,47	-	-
**2 Gal, 3 Man, 1 Fuc, 2 NeuAc, 6 GlcNAc**	3414	-	-	1,67
**4 Hex, 3 Man, 1 NeuAc, 2 Fuc, 3 HexNAc, 2 GlcNAc/ 3 Hex, 3 Man, 3 Fuc, 1 NeuGc, 3 HexNAc, 2 GlcNAc**	3432	1,57	-	-
**5 Gal, 3 Man, 5 GlcNAc, 1 Fuc, 1 NeuAc/4 Gal, 3 Man, 5 GlcNAc, 2 Fuc, , 1 NeuGc**	3462	6,07	4,27	-
**5 Gal, 3 Man, 5 GlcNAc, 1 Fuc, 1 NeuGc/6 Gal, 3 Man, 2 GlcNAc, 3 HexNAc, 1 NeuAc**	3492	3,13	4,11	-
**2 Gal, 3 Man, 4 Fuc, 5 HexNAc, 2 GlcNAc**	3501	-	-	1,20
**3 Gal, 3 Man, 1 Fuc, 2 NeuAc, 6 GlcNAc**	3660	-	-	0,63
**4 Gal, 3 Man, 2 Fuc, 1 NeuAc, 6 GlcNAc**	3677	-	-	1,28
**7 Gal, 3 Man, 6 GlcNAc, 1 Fuc**	3754	1,71	3,50	-
**4 Gal, 3 Man, 1 Fuc, 2 NeuAc, 6 GlcNAc**	3863	-	-	1,68
**4 Gal, 3 Man, 1 Fuc, 3 NeuAc, 6 GlcNAc**	4226	-	-	0,92
